# Information-theoretical analysis of the neural code for decoupled face representation

**DOI:** 10.1371/journal.pone.0295054

**Published:** 2024-01-26

**Authors:** Miguel Ibáñez-Berganza, Carlo Lucibello, Luca Mariani, Giovanni Pezzulo

**Affiliations:** 1 IMT School for Advanced Studies, Lucca, Italy; 2 Istituto Italiano di Tecnologia, Napoli, Italy; 3 Institute for Data Science and Analytics, Bocconi University, Milano, Italy; 4 Department of Physics “E. R. Caianiello”, University of Salerno, Fisciano, Italy; 5 Institute of Cognitive Sciences and Technologies, National Research Council, Roma, Italy; TU Wien: Technische Universitat Wien, AUSTRIA

## Abstract

Processing faces accurately and efficiently is a key capability of humans and other animals that engage in sophisticated social tasks. Recent studies reported a decoupled coding for faces in the primate inferotemporal cortex, with two separate neural populations coding for the geometric position of (texture-free) facial landmarks and for the image texture at fixed landmark positions, respectively. Here, we formally assess the efficiency of this decoupled coding by appealing to the information-theoretic notion of description length, which quantifies the amount of information that is saved when encoding novel facial images, with a given precision. We show that despite decoupled coding describes the facial images in terms of two sets of principal components (of landmark shape and image texture), it is more efficient (i.e., yields more information compression) than the encoding in terms of the image principal components only, which corresponds to the widely used eigenface method. The advantage of decoupled coding over eigenface coding increases with image resolution and is especially prominent when coding variants of training set images that only differ in facial expressions. Moreover, we demonstrate that decoupled coding entails better performance in three different tasks: the representation of facial images, the (daydream) sampling of novel facial images, and the recognition of facial identities and gender. In summary, our study provides a first principle perspective on the efficiency and accuracy of the decoupled coding of facial stimuli reported in the primate inferotemporal cortex.

## 1 Introduction

Recognizing faces and facial expressions with high accuracy is central for many cognitive and social tasks that primates (and possibly other animals) perform every day [1]. Several studies reported single neurons in the ventral visual stream—and particularly in the so-called “face patches” of the inferotemporal (IT) cortex—that are exquisitely sensitives to faces [2, 3].

A recent landmark study greatly contributed to shed light on the neural code for facial identity in the IT of macaques [4]. This study reported that faces might be represented as feature vectors in a relatively low-dimensional (∼50D) *face space* [5], with IT neurons tuned to single axes of variation of the face space and insensitive to changes in other, orthogonal axes (see note 1 in the Supporting information). Interestingly, distinct subpopulations of neurons appear to project faces onto two distinct sets of axes, which encode the geometric *shape* of a face and its *texture* (at a fixed shape) separately. The shape coordinates describe the main facial proportions, whereas the texture coordinates bring information about the detailed form of facial soft tissues, the skin texture and tonality, and cues to the facial shape in the depth dimension, through the light reflection.

From a computational perspective, these findings suggest that the IT cortex might form a generative model in which shape- and texture-related information is *decoupled* into separate factors (aka disentangled or factorised). The resulting *decoupled coding* ($R_{D}$) resembles closely a computer vision model called the Active Appearance Model (AAM) [6]. A recent computational study indicates that the AAM provides a very good fit for the single cell IT data of [4], outperforming most standard deep network models of visual processing in the ventral stream [7]. While the deep networks achieve a high score in face (or object) recognition, they do so by *multiplexing* the same information into different neurons, which is the opposite of the decoupling strategy reported in IT neurons by [4]. In keeping with this, another computational study [8] showed that using a deep generative model (*β*-VAE), with the explicit objective of disentangling facial images into separate latent factors, provides a good account of IT neural firings [4].

In a series of neural network simulations [4, 7], the face processing performance of the decoupled coding ($R_{D}$) that emerges from IT recordings is compared with a simpler scheme that is standard in computer vision: *eigenface* ($R_{E}$) *coding* [5, 9, 10]. In both $R_{E}$ and $R_{D}$ codings, each neuron “projects” facial images linearly onto one axis of variation of the face space. However, the projection is different in the two. In $R_{E}$ coding, the neurons simply encode the projection of the input face into the axes of variation of the original set of known facial images. Rather, in $R_{D}$ coding, the input facial image is first divided into two sources of information: a (shape-free) average-shaped or *uniformed* facial image whose texture corresponds to that of the input face, and a (texture-free) vector of Cartesian coordinates of some facial reference points called *landmarks*, describing the input face shape. Then, in $R_{D}$ coding, one set of neurons encodes the linear projection of the input *uniformed* facial image on the axes of variation *of uniformed images*, whereas another set of neurons encodes the projection of the input vector of landmark coordinates on the axes of variation of vectors of landmark coordinates. As reported in [4], the decoupled coding scheme $R_{D}$ explains a significantly higher fraction of neural data variance than the $R_{E}$ coding.

While the above studies assess that decoupling information is a key ingredient of facial processing in primates, it is still unclear why this is the case. A plausible formal rationale for the decoupling of shape and texture parameters (as done in the AAM and related models) is that they might vary independently in real life conditions. For example, small variations in facial expressions entail a significant change of shape but not texture, whereas different conditions of luminosity and age may induce significant variations in texture but not shape [4]. This line of reasoning leads to the untested idea that decoupled coding entails not just a more *accurate* but also a more *efficient* (or compact) description of facial data.

Indeed, various normative principles have been proposed that characterize the *efficient coding* of data from a source in terms of information demands [11–13] (see references in [14]). From an information theoretic perspective, a formal measure of code efficiency is its *description length*: the best model is the one that minimizes the amount of information (bits) required to encode both the data, in terms of the model’s latent variables, and the model parameters themselves [15–17]. This implies that a more complex model, which has more free parameters and requires more memory to be encoded, will only outperform a simpler model if it affords significantly more data compression—which in turn requires that it captures well the statistical structure of the data.

Here we use the notion of efficient coding to ask whether, why, and in which conditions the neural code for face representation found in monkey IT neurons, which is based on texture-shape decoupling ($R_{D}$ coding), is more efficient than a simpler description in terms of principal components of facial images, without texture-shape decoupling (eigenface coding $R_{E}$). For this, we compare the description length of (the principal components of) the two elements of $R_{D}$ coding—namely, shape-free texture and shape coordinates—with the description length of (the principal components of) the original facial images, using the same stimuli dataset as in the monkey study of [4].

Our main contributions are the following. We show that the neural code based on texture-shape decoupling ($R_{D}$) is more efficient than the eigenface coding ($R_{E}$). This is because, on the one hand, storing the principal components of few (significant) landmark coordinates comes at the cost of *little extra information* but, on the other hand, the uniformed (shape-free) facial images require less information to be encoded with the same precision. Indeed, the uniformed facial images present significantly larger inter-pixel correlations, with respect to the original set of facial images, and can consequently be described using fewer principal components. The information that is saved in the uniformation process may exceed the information needed to encode the shape coordinates, hence yielding an overall positive information gain in favour of $R_{D}$. Furthermore, our results reveal that such advantage of the decoupled coding increases with image resolution and when encoding variants of training set images that differ for facial expressions. This result is interesting, as it shows that the decoupled coding is most effective in a condition that is frequent in social cognitive tasks, such as the identification of variants of known faces (in expression, in luminosity conditions, or in age). Finally, to further consolidate our findings, we show that decoupled coding leads to a higher efficiency in a range of cognitively relevant tasks, which include the daydream generation of novel faces, the synthesis of unknown faces, and the recognition of facial identities and gender.

## 2 Materials and methods

### 2.1 Database

In our analysis, we use the FEI dataset [18, 19], which was also used in the characterisation of the neural code of facial identity in macaques [4]. The FEI dataset comprises *N* = 400 b/w pictures of dimension *w*_max_ × *h*_max_ = 250 × 300 pixels, accompanied by the spatial coordinates of *n*_ℓ_ = 46 standard landmarks for each image.

### 2.2 Texture and shape coordinates

Let the training set consist of *N*_tr_ facial images, $I={\left\{I\left(n\right)\right\}}_{n=1}^{N_{\text{tr}}}$, where **I**(*n*) is the *n*-th image, and of *N*_tr_ vectors of shape coordinates $L={\left\{\ell \left(n\right)\right\}}_{n=1}^{N_{\text{tr}}}$, where ℓ(n) is the vector of shape coordinates characterising the geometry of the *n*-th facial image. All images are vectors $I\left(n\right)=(I_{1}\left(n\right),\ldots ,I_{d_{t}}\left(n\right))$ of dimension *d*_t_ = *w* × *h*, where *w*, *h* are the width and height of the images in pixels (grid spacing units). The ***ℓ***-vector components are the x or y Cartesian coordinates of *n*_ℓ_ representative landmarks of the *n*-th facial image: $\ell \left(n\right)=(\ell_{1}\left(n\right),\ldots ,\ell_{d_{s}}\left(n\right))$, with *d*_s_ = 2*n*_*ℓ*_.

### 2.3 Formal definitions of eigenface coding ($R_{E}$) and decoupled coding ($R_{D}$)

**Fig 1:**
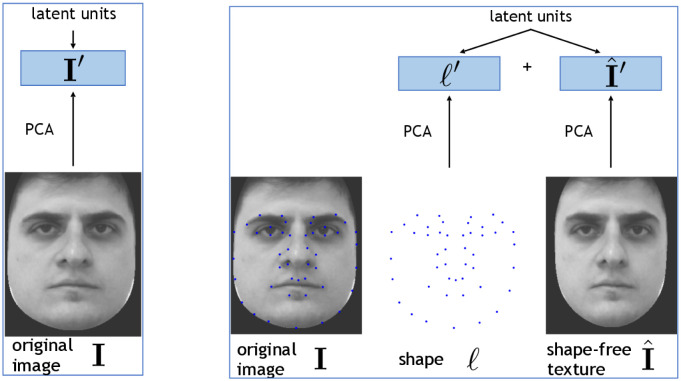
Schematic illustration of eigenface coding ($R_{E}$, left) and decoupled coding ($R_{D}$, right). See the main text for explanation. The two leftmost images are republished from [19] under a CC BY license, with permission from Carlos Eduardo Thomaz, original copyright 2006.

We consider two alternative neural codes for facial images: *eigenface* ($R_{E}$) *coding* and *decoupled* ($R_{D}$) *coding*; see Table 1 and Fig 1. Both the $R_{D}$ and the $R_{E}$ codings represent facial images in terms of Principal Components (PCs), but over different facial coordinates of the training set (i.e., over different datasets). Specifically, they represent a generic image **I** as follows:

1. *Eigenface coding* ($R_{E}$) represents the image in terms of its PCs, **I**′. In mathematical terms, $I^{\prime }=E_{p}^{\left(E\right)}\cdot I$, where $E_{p}^{\left(E\right)}$ is the $p\times d_{t}$ matrix composed of the first *p* (row) eigenvectors of the unbiased estimator of the correlation matrix *C* of training-set images, *C*_*ij*_ = 〈*I*_*i*_(*n*)*I*_*j*_(*n*)〉, where $\langle \cdot \rangle =\left(1/N_{\text{tr}}\sum_{n}\cdot \right)$ is the empirical average over the training-set, and where all the vector components are null-averaged, 〈*x*_*i*_〉 = 0. This representation does not make use of the shape coordinates.
2. *Decoupled coding* ($R_{D}$) represents the image in terms of two sets of PCs, one for shape and one for texture facial coordinates. To obtain these coordinates, each original image **I**(*n*) in the training set is first deformed by means of image-deformation algorithms (see [20–22] and the Supporting information for details), in such a way that its landmark coordinates ***ℓ***(*n*) are dragged to the *average position of the landmark coordinates in the training-dataset*, and that the rest of the image pixels are deformed coherently (so that the resulting facial image is as much realistic as possible). The resulting image will be called the *uniformed* image $\hat{I}\left(n\right)$ (see Fig 1). We refer to *uniformed texture coordinates*, or simply *texture coordinates*, as the *uniformed* (shape-free) image coordinates $\hat{I}$, of an image **I** given ***ℓ*** (and the average position of the landmarks 〈ℓ〉=0). This procedure permits decoupling the original dataset in two datasets of coordinates: the (texture-free) shape coordinates L and the (shape-free) uniformed images $\hat{I}={\left\{\hat{I}\left(n\right)\right\}}_{n=1}^{N_{\text{tr}}}$.
  The novel image **I** to be represented is then decomposed in PCs in texture and shape spaces separately, ${\hat{I}}^{\prime }=E_{p}^{\left(t\right)}\cdot \hat{I}$, $\ell^{\prime }=E_{p}^{\left(s\right)}\cdot \ell$, where $E_{p}^{\left(t\right)}$ are the eigenvectors of $C_{ij}^{\left(t\right)}=\langle {\hat{I}}_{i}\left(n\right){\hat{I}}_{j}\left(n\right)\rangle$, and $E_{p}^{\left(s\right)}$ those of $C_{ij}^{\left(s\right)}=\langle \ell_{i}\left(n\right)\ell_{j}\left(n\right)\rangle$.

**Table 1:** Outline of the two alternative schemes for the representation of facial images: Eigenface coding ($R_{E}$) and decoupled coding ($R_{D}$).

Code							
$R_{E}$	(**I**,***ℓ***)			$\overset{\text{projection}}{\to }$	$I_{p}^{\prime }=E_{p}^{\left(e\right)}\cdot I$	→	$\left(I_{p_{s}}^{\prime }\right)$
$R_{D}$	(**I**,***ℓ***)	$\overset{\text{uniformation}}{\to }$	$\left(\hat{I},\ell \right)$	$\overset{\text{projection}}{\to }$	$\begin{array}{l} {\hat{I}}_{p}^{\prime }=E_{p}^{\left(\text{t}\right)}\cdot \hat{I}\\ \ell_{p}^{\prime }=E_{p}^{\left(\text{s}\right)}\cdot \ell \end{array}$	→	$\left({\hat{I}}_{p_{s}}^{\prime },\ell_{p_{t}}^{\prime }\right)$

### 2.4 Description length analysis

#### 2.4.1 Intuition behind the description length analysis

We here provide an informal introduction to the concept of description length and its relation to Bayesian statistics. The notation is summarised in Table 2.

**Table 2:** List of symbols.

symbol	definition	name
D	{**x**(*n*)}_*n*_	generic dataset
$P_{M}\left(x|\theta \right)$		likelihood of vector x according to model M
$P_{M}\left(D|\theta \right)$		likelihood of D
$P_{M}\left(D\right)$	$=\int d\theta P_{M}\left(\theta \right)P_{M}\left(D|\theta \right)$	Bayesian evidence of D
$L_{M}\left(D\right)$	$=-{\text{log}}_{2}(P_{M}\left(D\right){∊}^{d})$	description length of D associated with M
$S(D|\theta^{*})$	$-{\text{log}}_{2}P_{M}\left(D|\theta^{*}\right)-d{\text{log}}_{2}∊$	empirical entropy of D
*O*(θ*)	$=L_{M}\left(D\right)-S\left(D|\theta^{*}\right)$	Occam factor of D

**Compression in PCA.** The Principal Component Analysis representation of a given set of coordinates in the face space with *p* principal components (*p*-PCA) can be viewed both as a generative model, inducing a Gaussian distribution over facial coordinates, and as a form of data compression and dimensionality reduction [23]. These two aspects are naturally linked by the notion of *description length* [17]. PCA is a form of dimensionality reduction, since it describes each *d*-dimensional vector **x** as a shorter, *p*-dimensional vector $x_{p}^{\prime }=E_{p}\cdot x$. In turn, this implies a *compression* ability. Consider a dataset in which each coordinate *x*_*i*_ (say, each pixel value, if the vectors **x** are images) varies uniformly in a range *R*. In the absence of any prior knowledge regarding the dataset content, the amount of information per sample and coordinate needed to store the raw dataset with precision *ϵ* per coordinate is simply *l*_0_ = log_2_(*R*/*ϵ*) bits. Normally, the information needed to store the *p* principal components of each vector of the dataset $D^{\prime }={\left\{x_{p}^{\prime }\left(s\right)\right\}}_{s=1}^{N}$ is lower than *l*_0_, even if *p* = *d*. Indeed, if the dataset exhibits significant pairwise correlations between couples of variables, several principal components will exhibit a variance λ_*i*_ lower than the average variance *R*^2^/12, and they will consequently require fewer bits to be stored. As we will see below, the overall information cost in the presence of pairwise correlations is, indeed, lower.

**Definition of description length.** If M is a probabilistic model over a set of vectors, generically called **x**, there exist (under conditions specified by the Kraft-McMillan theorem [17, 24, 25]) a *code* $x\to \tilde{x}$ such that any original vector **x** can be recovered from $\tilde{x}$ without losses, and such that the length in bits of each code vector $l\left(\tilde{x}\right)=-{\text{log}}_{2}\left(P_{M}\left(x\right)\right)$, where $P_{M}\left(x\right)$ is the probability of **x** according to M. The amount of information necessary to encode a dataset D={x(1),…,x(N)} in terms of the code associated to the probabilistic model M is called *description length*, $L_{M}\left(D\right)=\sum_{n=1}^{N}l\left(x\left(n\right)\right)$. In the context of Bayesian inference, and assuming that the **x**’s are vectors of real numbers, the description length $L_{M}\left(D\right)=-{\text{log}}_{2}\left[P_{M}\left(D\right){∊}^{d}\right]$, where $P_{M}\left(D\right)$ is the data evidence, or joint marginal likelihood of the dataset D according to M, and *ϵ* is the precision per coordinate with which the dataset should be described. The Minimum Description Length principle prescribes that the best model M for the data D should be chosen as the one exhibiting a lower description length.

**Intuitive view of description length.** The Bayesian evidence of the data $P_{M}\left(D\right)$ can be defined as the marginal over the set of parameters ***θ*** of the data likelihood given ***θ***, times the prior over ***θ***: $P_{M}\left(D\right)=\int d\theta \;P\left(D|\theta \right)P\left(\theta \right)$ (from now on we omit the model label M). Performing a second-order expansion of the data likelihood around its maximum value, the logarithm of the Bayesian evidence of the data can be written as ${\text{log}}_{2}P\left(D\right)={\text{log}}_{2}P\left(D|\theta^{*}\right)-O\left(\theta^{*}\right)$ where $P(D|\theta^{*})$ is the *dataset maximum likelihood* according to the probabilistic model *P*(⋅|***θ***), and *O*(θ*) is related to the determinant of the Fisher matrix, that accounts for the sensitivity of the data likelihood with respect to variations of the parameters around the maximising value at θ*. The description length can be consequently decomposed in two terms, $L\left(D\right)=S(D|\theta^{*})+O\left(\theta^{*}\right)$, that we will call, respectively, the *empirical entropy* $S\left(D|\theta^{*}\right)=-{\text{log}}_{2}P\left(D|\theta^{*}\right)-d{\text{log}}_{2}∊$ and the *Occam length* *O*(θ*). Again, to each likelihood probability distribution $P(x|\theta^{*})$ of the data vectors, it is associated a compressing code $x\to \tilde{x}$, that exploits the statistical regularities of the data according to the model *P*(⋅|***θ***), and such that the length in bits of the database D when encoded according to such a code is $S(D|\theta^{*})$. Note that the length in bits of the original database is simply *Nd*log_2_(*R*/*ϵ*), which is precisely the description length associated to a *uniform* probability distribution, with a constant density $P\left(x\right)=1/R^{d}$. A lower bound to the empirical entropy of the database corresponds, for large dataset lengths, to the lowest number of bits with which the database can be compressed (using the *best* code) and, if the dataset vectors have been sampled independently from a probability distribution $P^{v}$, this bound is the entropy of the distribution $P^{v}$. In this case, and if *N* is high enough, the higher the data likelihood, the closer the probability distribution *P*(⋅|θ*) is to $P^{v}$, and the lower is the empirical entropy.

Description length is therefore equivalent to—and provides an information-theoretic interpretation of—Bayesian model evidence. For large enough *N*, the Occam factor is negligible in front of the empirical entropy (which is intensive in *N*), and the model presenting minimum description length is that presenting a maximum likelihood $P_{M}\left(D|\theta^{*}\right)$, or a minimum empirical entropy. If, instead, *O*(θ*) is non-negligible in front of $S(D|\theta^{*})$, the model exhibiting a higher Bayesian evidence (or a lower description length) is the one presenting an optimal accuracy/complexity trade-off. In other words, description length analysis evaluates the efficiency of a particular code, taking into account both its accuracy and its complexity, which is penalised by the Occam factor, since complex models are more sensible to parameter tuning around θ* and, consequently, present larger curvature of lnP(D|θ) around θ*, or larger *O*(θ*). In this perspective, a good code is the one that does not employ too much information to describe a given input with a given tolerance. Indeed, the model that presents lower description length at fixed precision, is also the one that manages to describe the dataset with a smaller error, ${\text{log}}_{2}∊=-\left({\text{log}}_{2}P_{M}\left(D\right)+L\right)/d$, when the amount *L* of available storing information is fixed.

**Description length of PCA.** In this study, the models M at hand are the *p*-component PCA (for various values of *p*) for which an explicit expression for P(D) exists (see section 2.4.3). In this case, the parameters θ* are the eigenvector matrix *E*_*p*_ and the vector of averages μ (maximising the likelihood of a training set $D_{\text{tr}}$ that may be different from the database D of which we evaluate the description length). The empirical entropy and the Occam factor may be interpreted, respectively, as: the amount of information needed to encode, without losses (see note 2 in the Supporting information), the dataset D in terms of the *p* principal components of each vector; and the model parameters θ* once for all the vectors (that are needed to recover each vector **x** from its principal components $x^{\prime }=E_{p}\cdot x$). When increasing the number of fitted principal components *p*, the empirical entropy of the training dataset decreases (see note 3 in the Supporting information) but the Occam length generally increases, since more eigenvectors *E*_*p*_ must be stored –and they may need to be stored with a higher precision, if the Occam length *per degree of freedom* increases. Over-fitting occurs when this balance is no longer worth, and the description length increases for increasing *p*. The larger *N*, the larger is S in front of O, and the larger is the value of *p* minimising the description length.

#### 2.4.2 Definition of the *information gap* criterion for the comparison of decoupled coding and eigenface coding

We measured the description length (in bits) of the alternative coding schemes $R_{E}$ and $R_{D}$ of facial images **I** that belong to a set of known images that have been used to train the model (training set) and to a set of unknown images that have not been used to train the model (test set).

Eigenface coding ($R_{E}$) encodes the original images **I** in terms of their principal components. We denote the description length associated to the compression of a dataset I according to $R_{E}$ as $L_{I_{\text{tr}},p}\left(I\right)$. The two sub-indices of *L* specify the model; they are, respectively, the training set with which the model parameters have been trained (see note 4 in the Supplementary information), and the value of *p*. This information completely determines the *p*-PCA model.

Decoupled coding $R_{D}$ describes each facial image **I** in terms of the principal components of (shape-free) uniformed texture and (texture-free) landmark coordinates ${\hat{I}}^{\prime }$, $\ell^{\prime }$. This decoupling is motivated by the hypothesis that shape-free uniformed images can be compressed more easily compared to the original images and their unknown variants; or in other words, that there is a parsimonious description of the dataset of uniformed faces $\hat{I}$, when represented in terms of their principal components ${\hat{I}}^{\prime }$. However, since decoupled coding ($R_{D}$) exploits both the texture and the shape coordinates (***ℓ*** and $\hat{I}$) of each facial vector, it has to store both sets of principal components ($\ell^{\prime }$ and ${\hat{I}}^{\prime }$) to represent the original image. Moreover, it has to store the principal axes in both the space of texture and shape coordinates (see note 5 in the Supplementary information).

*The key question addressed here is whether the extra information cost required to store shape coordinates might be compensated by the smaller cost to store the uniformed texture principal components ${\hat{I}}^{\prime }$*. In principle, the uniformed set of images $\hat{I}$ might be compressed more easily, given that the inhomogeneities induced by the difference in landmark positions have been removed from the dataset—at least, if the resolution of the image is large enough. This implies that encoding the uniformed images could in principle require a smaller number of PCs without loss of precision, with respect to the set of raw images.

To quantify the difference in description length between uniformed texture coordinates, non-uniformed texture coordinates, and shape coordinates, we define a summary measure that we call an *information gap* and which jointly considers two factors. The former factor ($G_{1}$), the *texture information gap*, accounts for the difference in the description lengths of the non-uniformed and uniformed image datasets:

$$\begin{array}{ccc} G_{1}=L_{I_{\text{tr}},p}\left(I\right) & - & L_{{\hat{I}}_{\text{tr}},\hat{p}}\left(\hat{I}\right)\\ \begin{array}{l} \text{bits}\;\text{to}\;\text{compress}\;\text{the}\;\text{dataset}\\ \text{of}\;\text{non-uniformed}\;\text{images}\;I \end{array} & - & \begin{array}{l} \text{bits}\;\text{to}\;\text{compress}\;\text{the}\;\text{dataset}\\ \text{of}\;\text{uniformed}\;\text{images}\;\hat{I} \end{array} \end{array}$$ (1)

where $\hat{I}$ is the dataset composed by the uniformed facial images in I. Note that in both the description length terms, the model M is assumed to be *p*-PCA. In these equations, *p* may be taken as the optimal value according to Bayesian model selection, i.e., the value (say, p*) for which the description length of I is minimum, and the same for ${\hat{p}}^{*}$.

The latter factor ($G_{2}$) is the description length of the set of shape coordinates $L={\left\{\ell \left(n\right)\right\}}_{n}$:

$$\begin{array}{c} G_{2}=L_{L_{\text{tr}},p_{s}}\left(L\right) \end{array}$$ (2)

where L is the set of landmarks corresponding to the images I, and $L_{\text{tr}}$ to those in $I_{\text{tr}}$. In this way, $G_{2}$ represents the number of bits which are necessary to encode the set of landmarks L with a given, implicit precision *ϵ*, in terms of their $p_{s}$ principal components (extracted from the set $L_{\text{tr}}$).

The information gap combines these two factors ($G=G_{1}-G_{2}$) and measures the efficiency (in information-theoretic terms) of decoupled coding $R_{D}$ compared to eigenface coding $R_{E}$:

$$\begin{array}{c} \text{Information}\;\text{gap}\;\text{in}\;\text{favour}\;\text{of}\;R_{D}:\;G=G_{1}-G_{2} \end{array}$$ (3)

Decoupled coding can be considered more efficient if the information gap G is greater than zero. This occurs when the amount of information that is saved when encoding the set of uniformed images in terms of principal components, $G_{1}$, exceeds the amount of information needed to encode (again, in terms of principal components) the shape coordinates, $G_{2}$. More precisely: *the condition G>0 implies that the amount of information needed to store the principal components of the dataset $I_{\text{tr}}$ of raw facial images is larger than the information needed to store the associated datasets ${\hat{I}}_{\text{tr}},L_{\text{tr}}$ of uniformed images and their corresponding landmarks*.

Conversely, the condition G>0 also implies that, using the same amount of available information, the joint dataset ${\hat{I}}_{\text{tr}},L_{\text{tr}}$ can be decomposed with larger accuracy in terms of principal components, than the dataset $I_{\text{tr}}$ (see also the Supporting information).

Therefore, and in practice, our comparison boils down to computing and comparing the description length of *different datasets*: $\hat{I}$, I, L (the first one being computed from the last two), in terms of their principal components, i.e., using the same statistical model (*p*-PCA) for all the three datasets, *but with different covariance matrices* (inferred, respectively, from ${\hat{I}}_{\text{tr}}$, $I_{\text{tr}}$, $L_{\text{tr}}$) *and values of p*. We therefore assess the efficiency of the decoupled coding $R_{D}$ by considering the difference in description length between I and $\hat{I}$, and whether it compensates for the description length of L (see note 6 in the Supplementary information).

Note that, when we refer to *the information needed to encode the datasets*, we implicitly mean that *the precision ϵ* with which the databases should be described is, as we will explain in more detail in section 2.4.3, the smallest precision *ϵ* = 1 for both image and landmark coordinates. Let us assume that nor the uniformation $I\to ({\hat{I}}_{p_{s}},\ell_{p_{t}})$, nor the de-uniformation $({\hat{I}}_{p_{s}},\ell_{p_{t}})\to I$ (see the Supplementary material) procedures induce errors or image artifacts. Under this hypothesis, if the landmark coordinates are recovered without losses ∊_s_ ≤ 1, and the uniformed images are encoded with the smallest precision ∊_ut_ = 1, also the reconstructed, de-uniformed images will be described with an error ∊_t_ = 1. Using, hence, ∊_ut_ = ∊_u_ = ∊_s_ = 1, and under these hypotheses, it is fair to compare the three description lengths as in the definition of G.

#### 2.4.3 Precision of shape and texture coordinates and estimation of the description length

For the calculation of the description length $L_{D_{\text{tr}},p}\left(D\right)$ we exploit the analytical solution of the Bayesian evidence of a multivariate normal distribution [26]. Note that in the case of texture coordinates, that are strongly undersampled *N* ⪡ *d*_t_, it is essential to use such (asymptotically) exact expression, instead of its more common Bayesian Information Criterion approximation [17, 27], see the Supporting information. The training dataset and the number of principal components completely define the parameters of the inferred normal distribution $M=(D_{\text{tr}},p)$, whose Bayesian evidence $P_{M}\left(D\right)$ can be estimated analytically (see [26] and the formulae in the Supplementary information). The estimation of the description length depends, as said before, on a precision per coordinate *ϵ* with which the data should be described by the probabilistic model, $L_{M}\left(D\right)=-{\text{log}}_{2}\left(P_{M}\left(D\right)\right)-d\;{\text{log}}_{2}\left(∊\right)$. The second term in this equation is equivalent to the factor transforming the differential entropy of a continuous probability distribution into a genuine entropy [28–30] once the continuous variables have been binned in bins of of size *ϵ*.

In the case of texture coordinates, in which the vectors are images and the vector components *I*_*i*_ are 8-bit greyscale values in the range (0, 255), the natural cutoff value is ${∊}_{t}=1$. In the case of shape coordinates, the coordinates ℓ_*i*_ are integers varying in a range [1, *h*], so that the natural choice should be ∊_s_ = 1 for the largest resolution *h*_max_, and *h*_max_/*h* for lower resolutions *h* < *h*_max_ (since we change the coordinates resolution by scaling the largest-resolution coordinates as *ℓ*_*i*_ → (*h*/*h*_max_)*ℓ*_*i*_). Scaling the precision in this way, the empirical entropies of shape coordinates do not depend on the resolution (see also *Likelihood and evidence of shape coordinates* in the Supplementary information). We actually intentionally underestimate the shape coordinates’ cutoff and set ∊_s_ = 0.1(*h*_max_/*h*), so that we overestimate the description length of shape coordinates, in order to present (see the next section) a conservative estimation of the precision range in which the decoupled coding prevails.

## 3 Results

### 3.1 Results of the description length analysis

#### 3.1.1 Information gap for known facial images in the training set, at different resolutions

In this section, we analyse how the efficiency of the $R_{D}$ coding varies as a function of the resolution of the dataset images. We expect that the information gap increases with the resolution. If the resolution is so low that the distance between pixels (normalised to the image height, *h*^−1^), is of the same order of the typical deviation of landmark coordinates from their average, ${\langle \ell_{i}^{2}\rangle }^{1/2}$, the uniformation will not have an effect and consequently the $R_{D}$ code may not be worth in terms of description length. In the opposite situation, $h^{-1}⪡{\langle \ell_{i}^{2}\rangle }^{1/2}$, we expect a larger information gap.

**Fig 2:**
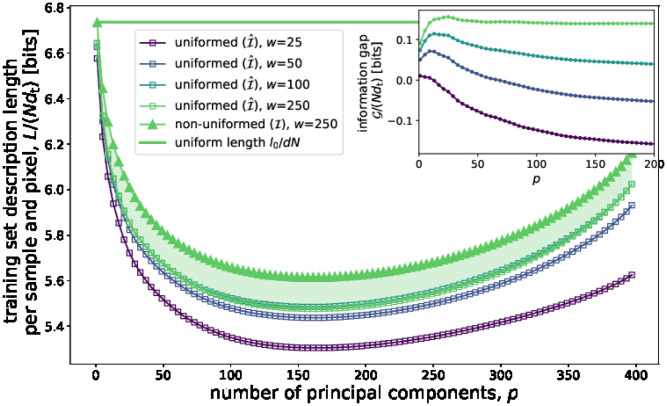
Description length of uniformed images in the training set. See the main text for explanation.

To test this hypothesis, we calculate p* for every kind of coordinate and resolution, as the minimum of the *L*_*p*_ curves. p* results to be lower than *N* in the three kinds of coordinates (shape, non-uniformed texture, uniformed texture). Fig 2 shows the description length of uniformed images in the training set (i.e., taking $\hat{I}={\hat{I}}_{\text{tr}}$ in Eq 1, where $\hat{I}$ is the whole dataset of *N* = 400 uniformed smiling and neural images) as a function of *p*, and for four different resolutions.

The description length of the image dataset is slightly over-linear in the number of pixels *d*_t_, as shown by the lack of superposition of the curves in Fig 2. Indeed, the largest images actually contain more information per pixel: this is the information that, according to the *p*-PCA model, has been lost when lowering their resolution to construct the lower-resolution datasets.

As a reference value for the analysis of description length curves, it is useful to compare the values in the figure with the *uniform length* *l*_0_/(*d*_t_*N*), or the minimum amount of information per sample and pixel that would take to store a dataset consisting in images whose pixels fluctuate *independently* around their average value in an interval of length *R*, being *R* such that the variance per pixel is equal to the empirical average variance ${\bar{v}}_{t}$ of the dataset I (roughly equal to 37 units per pixel out of 256 in 8-bit greyscale encoding, see note 7 in the Supplementary information). Fig 2 also shows the description length of non-uniformed images for the largest resolution, *w*_max_ × *h*_max_ = 250 × 300. We see that, for this resolution, the information gap in Eq 1 is positive: the uniformed images are better compressed than non-uniformed images, for all values of *p*. The information gap per sample and coordinate $G/(Nd_{t})$ is, as expected, an increasing function of the resolution—see the inset of Fig 2—indicating that the information gap increases faster than linear in *d*_t_. Rather, for the two lowest resolutions, decoupled coding does not lead to a gain in information. Indeed, for *w* = 25 the information gap is negative, roughly equal to minus one hundred of bits per sample.

**Fig 3:**
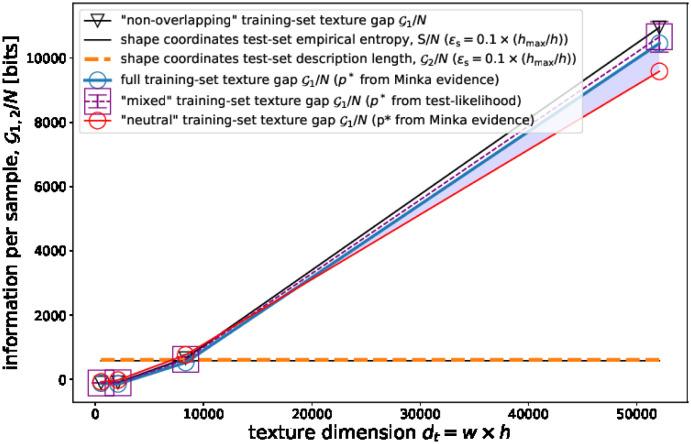
Information gap for facial images in the training set. See the main text for details.

The information gap per sample of the $R_{D}$ coding increases rapidly with the number of pixels *d*_t_, and it reaches more than 10000 bits per image for *w* = 250. This is evident in Fig 3, which shows the information gap per sample $G/N_{\text{tr}}$ of training set images as a function of the resolution. Fig 3 also shows the description length of shape coordinates, $L_{L_{\text{tr}},p_{s}^{*}}\left(L_{\text{tr}}\right)$, which is independent of the image resolution (horizontal line, see the details in the Supporting information). The information gap of the image degrees of freedom results comparable with the shape coordinates’ description length $L_{L_{\text{tr}},p_{s}^{*}}\left(L_{\text{tr}}\right)$ for *w* = 150, but it is much larger for the largest resolution *w* = 250. Summarising, for the largest image resolution, the texture-shape decoding entails a gain in information for the largest resolution. For *d*_t_ ≳ 10^4^, the condition in Eq 3 is satisfied.

A significant observation is that the standard deviation per pixel, ${\bar{v}}_{t}^{1/2}$, of the order of 37 units, is *not significantly smaller* in the dataset of uniformed images $\hat{I}$. This means that uniformed images are more easily compressible, not simply because the dataset is less-varying, or more homogeneous, but because of the presence of stronger pairwise correlations between pixels in the uniformed images. Stronger correlations induce a more inhomogeneous spectrum of *C*^(t)^, say $\lambda_{1}^{\left(t\right)},\ldots ,\lambda_{p}^{\left(t\right)}$ and, consequently, a lower empirical entropy S of the associated Gaussian distribution, which, up to an additive constant, depends on the eigenvalues (see the Supplementary information) as $\left(1/2\right)\sum_{i\leq p}\text{ln}\lambda_{i}^{\left(t\right)}$. Indeed, for the highest resolution, the excess of standard deviation per pixel and sample of non-uniformed images is ≃0.8. Neglecting the correlations, this would amount to an increment of *uniform length* $l_{0}/\left(d_{t}N\right)$ (or, equivalently, of $L_{0}/\left(d_{t}N\right)$) of ≃0.04, which is one order of magnitude lower than the texture gap $G_{1}$. This demonstrates that the differences in description lengths cannot be attributed to the reduction of total variance induced by the uniformation.

As a consistency analysis, we have calculated the information gap in two different ways: first, taking p* according to Bayesian model selection, $p^{*}=\text{arg}{\text{max}}_{p}L_{D_{\text{tr}},p}\left(D_{\text{tr}}\right)$; second, taking the value that maximises the validation-set (out-of-sample) likelihood, by *K*-fold cross-validation of the validation/training separation of the original dataset. Both ways of computing the training-set information gap are consistent within the (cross-validation) statistical errors (which is not obvious, specially considered that *d*_t_ ≫ *N*).

#### 3.1.2 Information gap for known facial images in the training set that show different facial expressions

In this analysis, we test the hypothesis proposed in the introduction that decoupled coding is particularly effective when encoding variants of known facial images which differ only in facial expressions. By definition, variations in facial expression are expected to change mainly shape coordinates, and much less texture coordinates (that are independent of the positions of the landmarks and hence nearly independent of the facial expression). The information gap should increase in this situation, since the texture coordinates of facial images differing in expression should be more redundant, correlated and easily compressed—or, in the language of probability, they should exhibit a larger likelihood.

To test this hypothesis, we computed the training-set information gap for two dataset of length *N*_tr_ = 200: the former (called “neutral”) consisting of neutral expression images of 200 different subjects and the latter (called “mixed”) corresponding to both the neutral and the smiling portraits of the same 100 (randomly selected) subjects. The blue shadowed area in Fig 3 indicates the difference in information gap between the “mixed” and the “neutral” training sets. While the information gap of the “mixed” dataset is indistinguishable from the “full” dataset of *N* = 400 images, the “neutral” dataset presents a lower information gap.

This analysis is consistent with our initial hypothesis. Notice that this result is not a trivial consequence of the fact that the “mixed” dataset (consisting in *N* = 200 portraits of *the same 100 subjects*) is more easily compressible than the “neutral” one (consisting in *N* = 200 portraits of 200 *different subjects*): indeed, for *both uniformed and non-uniformed* facial images, the description length of the “mixed” dataset is lower than that of the “neutral” dataset. What is less trivial is that *the gap G is higher for mixed images*.

#### 3.1.3 Information gap for unknown facial images in the test set that show different facial expressions

Here, we perform a variant of the above analysis aimed to test that the decoupling is particularly efficient when encoding *unknown* (not belonging to the training set) facial images that correspond to subjects that *are present* in the training set, with a different facial expression.

We have already seen that the training-set of uniformed images exhibits lower description length (and empirical entropy) than the training set of raw dataset images. It is hence reasonable to suppose that decoding does not only reduce the *bias error* (of the training-set) but also the *variance error* in the description of unknown facial images, belonging to a test-set (see note 8 in the Supplementary information).

To test this hypothesis, we calculated the information gap in a test-set I in Eq 2, which is composed by *N*/*K* = 80 (with *K* = 5) images corresponding to smiling subjects, *whose neutral-expression images do belong to the training-set*. Notice that we will call such a set simply “test-set”. All the information-theoretical quantities are then cross-validated for different *K* training/test partitions of the original dataset (by means of the *K*-fold algorithm of cross-validation).

**Fig 4:**
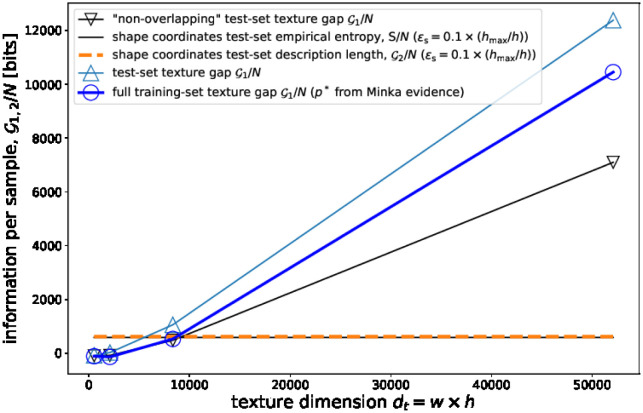
Information gap for facial images in the test set. See the main text for details.

Fig 4 reveals that the information gap of the test-set is significantly higher compared to the information gap of the training-set, with a p-value lower than 10^−4^ (notice the small error bars of the training-set information gap in Fig 3). The increment in information gap per sample (roughly 1/6 of the test-set gap) corresponds to the bits that one saves using the decoupled coding for unknown smiling faces, not belonging to the training-set. This implies, as expected, that the $R_{D}$ coding reduces both the bias and the variance errors for variants of known faces differing in facial expression only. $R_{D}$ *is, hence, particularly efficient*, in terms of information, *to encode facial images of known subjects, differing in facial expressions*.

It is interesting to compare this result with the texture information gap of a different test-set, which we call “non-overlapping”, in which the single folds are composed by *N*/*K* = 80 facial images corresponding to 40 subjects with both smiling and non-smiling expression. The non-overlapping test-set contains, in this way, images of subjects *that are not present in the training-set* (so that test- and training-sets contain information regarding different subject identities). Fig 4 shows how the texture information gap $G_{1}$ of the non-overlapping dataset is even lower than the training-set gaps. This result shows that the decoupling code $R_{D}$ *is less efficient to encode unknown facial images corresponding to unknown subjects*. Furthermore, this results shows that while uniforming variants of known faces that differ only in facial expression implies a reduction of both the bias and the variance terms of the entropy (see note 8 in the Supplementary information), uniforming facial images of unknown individuals leads to a reduction of the bias term *but to a positive increment of the variance term*. In any case, we stress that the texture information gap $G_{1}$ is still larger than $G_{2}$ for the non-overlapping test-set. Consequently, the decoupling code is more efficient even when processing unknown-identity facial images, albeit in this case the description length gap is lower.

#### 3.1.4 Summary of the results of the description length analyses

In sum, our analysis shows that decoupled coding leads to a more efficient encoding of known facial images (i.e., in the training set) compared to eigenface coding, when the images are shown at high enough resolutions and in particular when they differ in facial expressions. Furthermore, our results show that the efficiency of the decoupled coding is magnified when the task consists in encoding *unknown* variants of known faces differing in facial expression.

### 3.2 Analysis of the performance of decoupled and eigenface coding in face processing tasks

So far, we have used the normative construct of description length to assess the efficiency of decoupled coding. Here, we ask how the normative advantage of decoupled coding translates into a better performance in facial processing tasks and what are exactly the advantages. For this, we compare the performance of eigenface and decoupled coding in three face processing simulations that help illustrate the most important differences between the coding schemes; namely, (1) sampling artificial facial images from the learned generative model, (2) recognizing facial identity, and (3) reconstructing unknown faces. Please see the Supporting information for a supplementary (gender classification) simulation.

#### 3.2.1 Simulation 1: Sampling synthetic faces from the trained generative model

The generation of artificial faces is a widely used task in AI to demonstrate the quality of a learning algorithm or encoder. In this simulation, our goal is not to challenge the performance of mainstream machine learning approaches that use deep nets with millions of parameters [31–34], but rather to test the hypothesis that a very simple (20 degrees of freedom) linear model can generate realistic images, when it is based on decoupled coding.

Each PCA-based representation of the training set I induces a simple generative model of faces (see the Supporting information for details). In particular, $R_{E}$ induces a multivariate Gaussian distribution in the space of facial images. Rather, $R_{D}$, induces two separate Gaussian distributions over uniformed texture and shape coordinates, respectively. It is possible to create *synthetic* facial images by sampling from the respective probability distributions of $R_{E}$ and $R_{D}$. In the case of $R_{D}$, after sampling from both probability distributions, it is necessary to de-uniformise the sampled uniformed texture coordinates given the sampled shape coordinates (see the Supporting information for details about the de-uniformation procedure).

**Fig 5:**
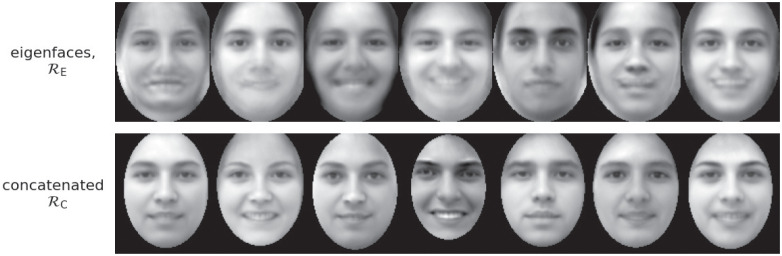
Examples of synthetic faces sampled from the generative models of $R_{E}$ (top row) and a simple variant of the decoupling $R_{D}$ coding scheme, which we call concatenated coding, $R_{c}$ (bottom row). See the main text for details.

Fig 5 shows example synthetic images created by sampling **I** and $\left(\hat{I},\ell \right)$ from the models induced by $R_{E}$ and $R_{D}$, respectively. In both cases, we used *p* = 20 degrees of freedom, randomly chosen among the first 40 principal components of each model (see note 9 in the Supplementary information). We sketch this procedure in see Algorithm 1.

**Algorithm 1** Sampling facial images algorithm with the $R_{c}$ code

1: **procedure** sample(L, $\hat{I}$; *p* = 20)

2:  $y\left(n\right)=(\hat{I}\left(n\right),\ell \left(n\right))$ concatenated variables for each picture *n* in the database

3:  $C_{ij}^{\left(\text{c}\right)}=\langle y_{i}\left(n\right)y_{j}\left(n\right)\rangle$, the $\left(d_{s}+d_{t}\right)\times \left(d_{s}+d_{t}\right)$ correlation matrix

4:  **for** *i* < *n*_samples_ **do**

5:   $y^{\prime }∼N\left(0,\Lambda \right)$ sample a vector of principal components

6:   (where Λ is the diagonal matrix of the 40 largest *C*^(c)^ eigenvalues)

7:   $y_{j}^{\prime }=0$ some (40 − *p*) out of the 40 principal components are set to zero

8:   $y=E^{†}\cdot y^{\prime }$ is the sampled concatenated vector

9:   (where *E* is the matrix with the row eigenvectors of *C*^(c)^)

10:   $(\hat{I},\ell ):=y$

11:   $(\hat{I},\ell )\to I$ de-uniformation

12:   **I** is the *i*-th sampled image

The larger the value of *p*, the higher the dimension of (the vector space of) the sampled facial images. When small values of *p* are used, the generative models produce low-dimensional variations of the average face; this implies that the synthetic faces are realistic (free from artefacts) but very stereotyped, with low variability. Rather, using larger values of *p* is a more compelling task, since the generative models are free to produce faces with high variability—but at the same time it is harder for them to produce realistic faces that are free from artefacts.

Fig 5 permits appreciating that with a relatively high value *p* = 20, both eigenface coding ($R_{E}$) and decoupled coding ($R_{D}$) produce produce faces with high variability. However, only the faces produced by the latter are realistic and free from artefacts. This simulation therefore shows that a very simple linear model based on decoupled coding (but not on eigenface coding) can produce realistic and varied facial images.

Please note that for this comparison we consider a simple variant of the decoupling $R_{D}$ coding scheme, which we call *concatenated coding*, $R_{c}$, consisting in the principal components of the concatenated set of texture and shape coordinates (see the Supporting information for a formal description). The reason is that $R_{c}$ permits choosing a single number of principal components, *p*, in common with $R_{E}$—which therefore permits comparing the two codes with the same number of parameters. The use of $R_{D}$ would require, instead, to choose separately $p_{t},p_{s}$ for a fixed $p_{t}+p_{s}=p$. Indeed, the results of Fig 5 are qualitatively identical if one directly compares $R_{E}$ with $R_{D}$, varying $p_{t}=p$ and fixing $p_{s}$ to its maximum value, $=d_{s}=92$). We remark that the concatenated coding is considered here and in the next subsection only for the sake of simplicity of the $R_{E}$-$R_{D}$ comparison (see note 10 in the Supplementary information).

#### 3.2.2 Simulation 2: Facial identity recognition

The results of the description length analysis show that decoupled coding may be particularly suited to encode efficiently natural variants of known facial images, which consist in variations of facial expressions. This is because variants of a known face may affect one set of coordinates shape or texture, while leaving the other essentially unaffected. For example, recognising a known face with a different facial expression might benefit from the reuse of uniformed texture coordinates, which would be relatively unaffected.

In this simulation, we test whether and how the description length advantage of decoupled coding translates into a better capability to recognise faces with different facial expressions. For this, we exploit the fact that the FEI dataset includes 400 facial images of 200 subjects, with 2 images of the each subject that only vary for facial expressions: neutral or smiling. The simulative task consists in recognising which of the 200 images showing a neutral expression corresponds to a target image (excluded from the training set) in which the same person shows a smiling expression, and vice-versa.

We implement the recognition task through a nearest-neighbour classifier, using a *distance* $d_{p}\left(z,x\left(s\right)\right)$ among the facial coordinates of the target smiling face z, and those of all the 200 neutral expression images x(s). The distance $d_{p}\left(\cdot ,\cdot \right)$ among facial coordinates is the Mahalanobis (+ *L*_2_) distance, known to be a better measure of facial similarity than the simple Euclidean metric between images [35, 36, Chs. 5 and 6] (see the note 11 in the Supplementary information). The Mahalanobis metrics between a couple of vectors is a scalar product between their (normalised) first *p* principal components (see note 12 in the Supplementary information), which does depend on the eigenvalues and eigenvectors of the correlation matrix and, hence, on the training set. It is important to remark that that the target image coordinates are excluded from the training set (see the details in the Supporting information, and in Algorithm 2).

**Algorithm 2** Error rate of identity recognition

1: **procedure** compute error rate(generic dataset D; *p*; *K*)

2:  **for** $D=(D_{\text{t}r},D_{\text{t}e})$ out of *K* training-test divisions of D **do**

3:   ($D_{\text{t}e}$ contains at most one vector corresponding to the same subject)

4:   ($D_{\text{t}e}$ contains $N_{\text{te}}/2$ corresponding to smiling subjects)

4:   $C_{ij}={\langle x_{i}x_{j}\rangle }_{x\in D_{\text{t}r}}$ training-set correlation matrix

6:   $d_{p}\left(u,v\right)={\left[{\left(u-v\right)}^{†}\cdot C_{p}^{-1}\cdot \left(u-v\right)\right]}^{1/2}$ define Mahalanobis distance

7:   **for** $x\in D_{\text{t}e}$ **do**

8:   find the $y\in D_{\text{t}r}$ such that $d_{p}\left(x,y\right)$ is minimum

9:   **if** **x**, **y** correspond to the same subject, *n*_success_+ = 1

10:  the success rate $r_{\text{success}}=n_{\text{success}}/\left(N_{\text{te}}K\right)$

11:  **return** error rate $r=1-r_{\text{success}}/N_{\text{tr}}$

**Fig 6:**
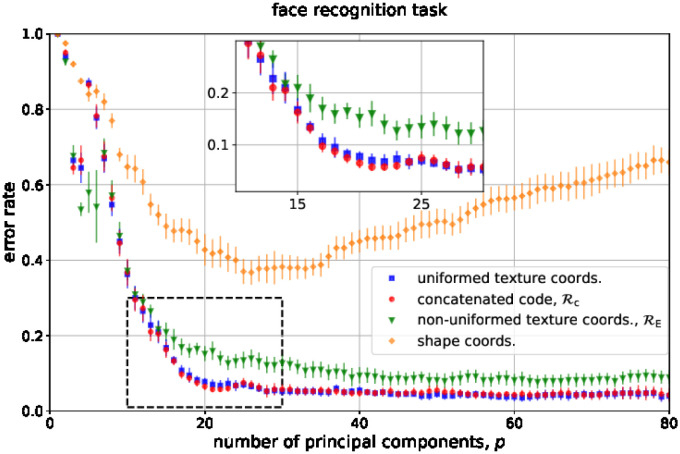
Performance of in the face recognition task using the uniformed images, the non-uniformed images, and the shape coordinates. See the main text for explanation.

Fig 6 shows the results of the facial identity recognition task. The figure reports the misclassification error as a function of the number *p* of principal components considered, for each kind of coordinate (uniformed images, non-uniformed images and shape coordinates, or x=I, $\hat{I}$, ***ℓ***, respectively). The errors decrease and reach a plateau in the cases of both non-uniformed and uniformed images, but the latter consistently exhibits a better performance. Interestingly, this simulation permits appreciating the relative contributions of texture and shape coordinates to the task. As expected, the performance of shape coordinates is significantly lower, with a minimum error around 0.4 (notice that in such recognition task, the chance level error rate is 1 − 1/*N*_train_ ≃ 0.996, see the Supporting information). However, we notice that this fact depends on the arbitrary choice of the image resolution (determining the image dimension *d*_t_ = *w* × *h*) and on the number of landmarks *n*_ℓ_ (determining the shape coordinate dimension *d*_s_ = 2*n*_*ℓ*_). Using a larger number of landmarks will enhance the relative relevance of shape coordinates. Moreover, Fig 6 reveals as well that the distance based on the concatenated code $R_{c}$, which exploits the correlation between shape and uniformed texture coordinates, does not perform significantly better than that based on uniformed images. This is due to the fact that the images contain more information than the shape coordinates (since $d_{t}\gg d_{s}$), and that shape and texture coordinates are only weakly correlated (see the Supporting information).

The reader may find a discussion on the qualitative differences in the shape of the error rate curves of texture and shape coordinates in the Supplementary information.

#### 3.2.3 Simulation 3: Reconstructing novel faces

The reconstruction task consists in representing novel facial images that do not belong to the training set, in terms of an expansion in *p* principal components only. In mathematical terms, if **x** is a facial image, or its shape coordinates, the reconstruction of **x** in terms of the first *p* principal axes is $x_{p}=E_{p}^{†}\cdot E_{p}\cdot x$, where *E*_*p*_ is the *p*×*d* matrix of the first *p* (row) eigenvectors (see note 13 in the Supplementary information). In the case of the $R_{E}$ code, we simply project the original image (x=I) onto the first *p* eigenfaces, $x_{p}=E_{p}^{†}\cdot E_{p}\cdot x$. In the case of $R_{D}$, we perform this operation for both ${\hat{I}}_{p_{s}}$ and $\ell_{p_{t}}$ (for $\left(\hat{I},\ell \right)=y_{p}$ in the case of $R_{c}$) and, afterwards, we perform a de-uniformation (see the Supplementary information, and Algorithm 3), leading to a non-uniformed reconstructed image $({\hat{I}}_{p_{s}},\ell_{p_{t}})\to I$ from the information present in both the reconstructed texture and shape coordinates.

Algorithm 3 Facial reconstruction with $R_{c}$

1: **procedure** compute error rate(target facial vector and landmarks (**I**,***ℓ***); *p*)

2: $I\to \hat{I}$ uniformation

3: $y=(\hat{I},\ell )$ concatenated variables

4: $y_{p}=E_{p}^{†}\cdot E_{p}\cdot y$ reconstruction

5:  (*E*_*p*_ is the matrix of the first *p* (row) principal axes)

6:  (computed from a training set to which y does not belong)

7: $\left({\hat{I}}_{p},\ell_{p}\right):=y_{p}$

8: $\left({\hat{I}}_{p},\ell_{p}\right)\to I_{p}$ de-uniformation

9:  **return** the reconstructed image **I**_*p*_

**Fig 7:**
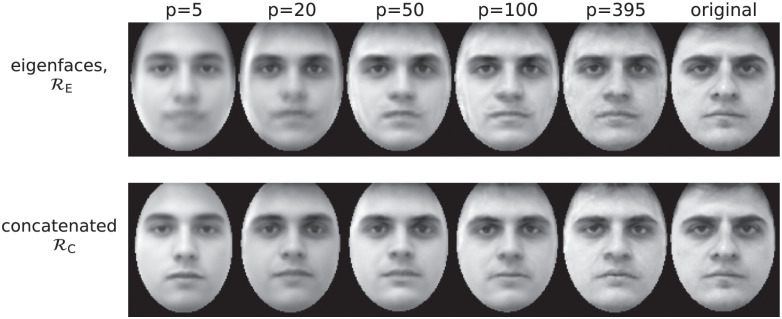
Reconstruction of a test-set facial image with *p* principal components according to the representations: Eigenface coding ($R_{E}$, first row) and concatenated coding ($R_{c}$, second row). The 5 columns of the matrix of images represent, respectively: *p* = 5, 20, 50, 100, 395, and the original image. The image is 100 × 120. The original image (rightmost column) is republished from [19] under a CC BY license, with permission from Carlos Eduardo Thomaz, original copyright 2006.

Fig 7 shows the reconstruction of a novel face according to $R_{E}$ and $R_{c}$ for different values of *p*. The figure illustrates that $R_{E}$ produces a border artifact; this is because linear combinations of different facial images with different facial contours result in an image which tend to be blurred in the margin of the face. Rather, the border artifact is absent for $R_{c}$, and the representation for high *p* is slightly more faithful.

**Fig 8:**
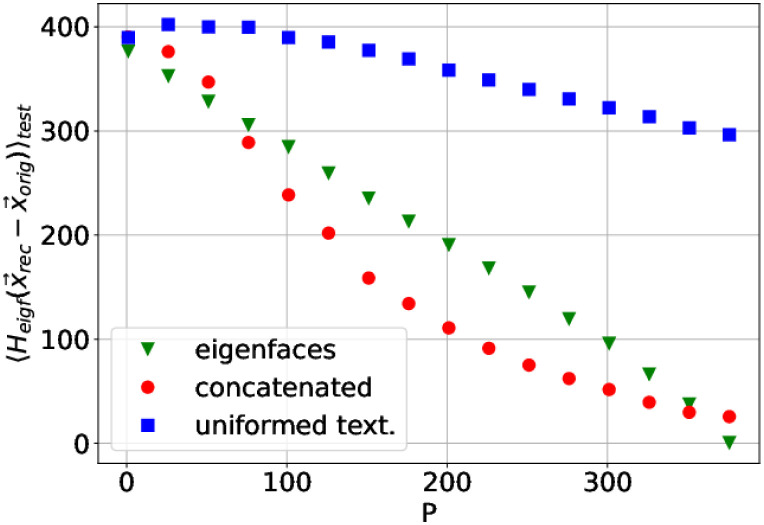
Mahalanobis distance $d_{C,p_{d}}\left({\text{t}}_{p},\text{t}\right)$ among target facial images and their reconstructed counterparts with *p* principal components (in the abscissa). The vectors **t**_*p*_ are reconstructed in terms of: eigenfaces (${E_{p}^{\left(\text{e}\right)}}^{†}\cdot I_{p}^{\prime }$, see Table 1); concatenated facial vectors (obtained by de-uniformation of $(\hat{I},\ell )\to I$, where $\left(\hat{I},\ell \right)=y_{p}={E_{p}^{\left(\text{c}\right)}}^{†}\cdot y_{p}^{\prime }$); uniformed texture components only ($\hat{I}$). The points are the average of the distance over $N_{\text{te}}=20$ randomly selected images with neutral facial expression. See the main text for an interpretation of these results.

This is quantitatively illustrated in Fig 8, which reports the Mahalanobis distance (see note 12 in the Supplementary information) between the target facial image, **t**, and its reconstruction from *p* principal components, **t**_*p*_, according to the representations $R_{E}$ and $R_{c}$. Crucially, in this analysis the target vectors are $N_{\text{te}}=20$ neutral, randomly chosen test-set images, *not belonging to the training set* from which the “reconstructing matrix” $E_{p}^{†}\cdot E_{p}$ is calculated. Also crucially, the matrix *C* defining the Mahalanobis distance $d_{C,p_{d}}\left(t_{p},t\right)=H_{C,p_{d}}\left(t_{p}-t\right)$ with $H_{C,p_{d}}\left(x\right)=x^{†}\cdot {C_{p_{d}}}^{-1}\cdot x$ is taken *as the (training-set) correlation matrix of the eigenface coding*, with the maximum number of principal components $p_{d}=N_{\text{tr}}-N_{\text{te}}-2=378$. Indeed, for a numerical assessment of the similarity between reconstructed and original faces, a perceptual distance based on a (dimensionality reduced) representation is required [5]. The simple pixel-wise Euclidean distance does not reflect correctly perceptual similarity and, indeed, we observed that it does not allow to assess any significant difference between the two representations. If, to compute the similarity between target and reconstructed images, one chooses a representation *equal to one of the representations used for the reconstruction* (the eigenface code $R_{E}$), we are biasing the comparison in favour of this representation. Despite the unfavorable bias, the $R_{c}$ code exhibits a lower perceptual distance in Fig 8 for all the values of *p* except for the maximum *p* = *p*M_d_ *for which, by construction, the $R_{E}$ code presents the minimum possible distance d=0* (since the reconstructed and the target image are expressed in terms of the same principal axes)*, and hence it is not possible that a different model improves it.* Significantly, for the rest of values of *p* < *p*_d_, the $R_{c}$ code improves $R_{E}$ despite such a bias.

For completeness, in Fig 8 we present the distance between target and reconstructed faces using the *uniformed* *p*-component images $\hat{I}$ according to the concatenated representation (i.e., omitting the de-uniformation step). In terms of the Mahalanobis metrics, the uniformed reconstructed images are less faithful than those based on the $R_{E},R_{c}$ codes. This demonstrates the importance of shape coordinates in the decoupled code for facial representation (see also the discussion on this point in sections 4).

Note that, for what concerns facial reconstruction, the decoupled code behaves exactly as the concatenated code. As for Simulation 1, the results of this subsection are qualitatively identical if one directly compares $R_{E}$ with $R_{D}$, varying $p_{t}=p$ and fixing $p_{s}$ to its maximum value $d_{s}=92$.

#### 3.2.4 Summary of the results of the face processing tasks

In sum, our analysis shows that a tiny model using decoupled coding and only 20 degrees of freedom can be sampled to produce realistic synthetic images, whereas sampling from a model using eigenface coding produces less realistic faces with artefacts. Furthermore, decoupled coding greatly facilitates the recognition of familiar faces with novel facial expressions—especially thanks the fact that texture coordinates remain stable across different expressions. Finally, decoupled coding outperforms eigenface coding in the reconstruction of novel faces. Please see the Supporting information for an additional simulation of gender classification using the two coding schemes.

Please, note that we release the codes with which we have performed the face processing tasks in the repository [37].

## 4 Discussion

Recent research in neuroscience claims that the neural coding for facial identity in the inferotemporal (IT) cortex of macaques [4, 7] implements a form of decoupled representation in which distinct subpopulations of neurons project facial images onto two distinct sets of axes encoding the facial shape texture separately. From a computational perspective, decoupled coding affords accurate face processing, permitting the *linear* decoding of facial features from single cell responses, and it outperforms widely used schemes in vision research, such as eigenface coding [4, 7, 8].

In this article, we aimed to elucidate the normative reasons for this advantage, by appealing to the notion of *description length*, which permits quantifying the efficiency of neural coding schemes in information-theoretical terms. The general idea is that the best model is the one that minimizes the amount of memory (bits) required to encode both the data and the model parameters themselves [15, 16].

In particular, we analysed the description length of the decoupled coding ($R_{D}$), aka the Active Appearance Model (AAM), which encodes both the principal components of *uniformed* (shape-free) facial images and their shape (landmark) coordinates. At difference with previous studies that compared alternative (biologically plausible) neural codes [4, 7], here we performed an information-theoretic analysis of the $R_{D}$ code only. In particular, we evaluated the gain in information entailed by the uniformation of facial images, and compared it with the amount of information required to encode the landmark coordinates. This evaluation necessarily requires calculating the information length of the principal components of non-uniformed images (i.e., that of eigenfaces) and, hence, it results in an implicit comparison of decoupled coding ($R_{D}$) and eigenface coding ($R_{E}$).

Our simulations, on the same dataset (FEI, see [19]) as in the monkey study of [4], show that decoupled coding ($R_{D}$) requires less information to represent the images compared to eigenface coding ($R_{E}$), despite the latter does not require coding for the geometric coordinates of faces. Remarkably and intuitively, the efficiency gain of decoupled coding ($R_{D}$) is especially prominent for high resolution images and for variants of training set images that only differ in facial expressions.

Furthermore, we found that the probabilistic generative model induced by decoupled coding ($R_{D}$) achieves good performance in face processing tasks, including sampling artificial or novel faces, recognising face identity and reconstructing novel faces with *p* principal components. Rather, a model using eigenface coding ($R_{E}$) performs less accurately and produces less realistic faces with artefacts.

The number of landmarks *n*_ℓ_ or, equivalently, the dimension of the shape coordinates (*d*_s_ = 2*n*_*ℓ*_) is, by construction, much lower than the image resolution *d*_t_. In information theoretical terms, it is precisely this condition *d*_s_ ⪡ *d*_t_ that makes the decoupled coding efficient: encoding the position of few landmarks costs an extra information which is much lower than the information that is saved when encoding the resulting *uniformed* facial images (for high enough image resolution). Indeed, a control calculation shows that this advantage is in place up until the landmarks become too numerous, i.e., of the order of a few hundreds (see the Supplementary information, paragraph “How many landmarks are too many?”).

We have seen that, in our analyses, the description length of the decoupled and concatenated codes almost coincide, and the contribution of shape coordinates to the facial recognition task is lower than that of images. This is because the number of landmarks is much lower than the number of pixels. However, this must not induce the reader to think that the decoupled (or the concatenated) code can be explained almost solely by texture coordinates, or that the overall information carried by shape coordinates is negligible. Indeed, the description length *per coordinate* of both kinds of coordinates, images and landmarks, is comparable (see Fig 2-main text and 2-Supporting information). More importantly, the facial reconstruction task reveals (Fig 8) how important are shape coordinates in the generative model that the $R_{D}$ induces *over the space of non-uniformed images*. This result illustrates how accuracy in the landmark coordinates is crucial to accurately represent a target facial image in terms of its decoupled principal components.

## 5 Conclusions

Taken together, these results shed light on the normative advantages of the decoupled coding for faces that was empirically reported in monkey inferotemporal (IT) cortex of macaques [4, 7], showing that it is more efficient (in info-theoretic terms) than the alternative eigenface scheme widely used in computer vision.

The dataset of uniformed facial images does *not* present, as one may have expected, significantly lower variance than the set of non-uniform images. The present information-theoretical analysis highlights that the efficiency of the decoupled coding comes, instead, from the fact that pixels of uniform images are more correlated and, consequently, they can be compressed in terms of few principal components without loss of precision. The amount of information saved in encoding uniform images compensates, for high enough resolution (and low enough number of landmarks), the amount of information needed to encode the principal components of shape coordinates.

*Shape* (texture-free) and *texture* (shape-free) coordinates carry information regarding naturally different aspects of human faces and can vary independently. Namely, variations in facial expressions and variations in perspective (e.g., small rotations) mainly modify shape coordinates, but not texture coordinates. Rather, variations in luminosity, suntan, or make-up only modify texture coordinates [38]. This perspective helps explain our finding that decoupled coding is particularly advantageous when encoding *variants of known faces* or unknown faces (in the test set). When encoding variants of known images, one of the two sets of (shape and texture) coordinates will tend to remain the same as in the known, reference image.

Of note, the above described advantages of decoupled coding come at the price of performing some prerequisite nonlinear computations over facial images: at least landmark detection and image deformation (see the Supporting information). We speculate that if the neural code for facial identification implements a variant of the AAM [4], these nonlinear computations might be putatively realised by early visual areas, which lie below the IT in the neural hierarchy. This speculation remains to be tested in future research, since the neuronal mechanisms of landmark detection and image deformation have not been identified yet. As a further future enhancement of this work, we propose to perform an analysis of the likelihood as a function of the number of landmarks; this would help improving our estimation of the optimal number of landmarks in the decoupling scheme, given the texture resolution (see *How many landmarks are too many?* in the Supporting information). Finally, future research could address the conditions under which the decoupling emerges spontaneously in deep architectures, as well as a study of the AMM efficiency in which the cost of landmark detection and uniforming is taken into account.

## Supporting information

S1 File The Supporting information document contains detailed information regarding protocols and mathematical methods. It is divided in the sections: *Notes*; *Relation with Bayesian Model Selection*; *Image uniformation and de-uniformation*; *How many landmarks are too many?*; *Likelihood and evidence of the normal distribution*; *Likelihood and evidence of shape coordinates*; *Likelihood and evidence of texture coordinates*; *The concatenated code representation*; *Details of the classification algorithms*; *Results of the gender classification task*; *Different regularisation schemes*; *Visualisation of the eigenvectors of the concatenated code*.
(PDF)
