# Supplementary material for: Information-theoretical analysis of the neural code for decoupled face representation
Source: PLoS One. 2024 Jan 26;19(1):e0295054. doi: 10.1371/journal.pone.0295054 (PMC10817192; doi:10.1371/journal.pone.0295054)
Supplement: S1 File — It is divided in the sections: Notes; Relation with Bayesian Model Selection; Image uniformation and de-uniformation; How many landmarks are too many?; Likelihood and evidence of the normal distribution; Likelihood and evidence of shape coordinates; Likelihood and evidence of texture coordinates; The concatenated code representation; Details of the classification algorithms; Results of the gender classification task; Different regularisation schemes; Visualisation of the eigenvectors of the concatenated code. (PDF) [file pone.0295054.s001.pdf]

Information-theoretical analysis of the neural code for decoupled face representation

SUPPORTING INFORMATION

Miguel Ibáñez-Berganza[1,2‡], Carlo Lucibello[3‡], Luca Mariani[4‡], Giovanni Pezzulo[5‡*]

1IMT School for Advanced Studies, Lucca, Italy
2Istituto Italiano di Tecnologia, Napoli, Italy
3 Institute for Data Science and Analytics, Bocconi University, Milano, Italy
4 Department of Physics "E. R. Caianiello", University of Salerno, Fisciano, Italy
5 Institute of Cognitive Sciences and Technologies, National Research Council, Roma, Italy

‡These authors contributed equally to this work.
* giovanni.pezzulo@istc.cnr.it

1 Supporting information

Notes

1. Indeed, neurons are believed to encode principal components *linearly* but not necessarily one-to-one, see [1]. In particular, if $\mathbf{y}$ is the vector of neurons' normalized firing rates and $\mathbf{x}'$ is the vector of principal components in the face space, an orthogonal matrix O relates $\mathbf{y}$ and $\mathbf{x}'$: $\mathbf{y} = O\mathbf{x}'$.

2. Crucially, the p-PCA model induces a (Gaussian) probabilistic model defined on the d-dimensional linear space of the data also when $p < d$. The $d-p$-dimensional subspace not expanded by the p fitted empirical eigenvectors (corresponding to the p largest eigenvalues λ_j) is described with a constant, degenerated noise eigenvalue $\bar{\lambda}$. As a consequence, despite the PCA representation of vectors in terms of p principal components *is a lossy representation* for $p < d$, the induced empirical entropy S term in the description length of each vector does *take into account the losses*, since it includes the information weight of the residuals of the approximation $\mathbf{x}' = E_p \cdot \mathbf{x}$.

3. Let the eigenvalues of the correlation matrix C of the training set be, in decreasing order, λ_i, with $i = 1, \ldots, d$. Then, given $p = 1, \ldots, d$, the empirical entropy is, up to an additive constant: $= (1/2)\sum_{i=1}^{p} \log_2 \lambda_i + (1/2)(d-p)\log_2 \bar{\lambda}$, being $\bar{\lambda} = (\sum_{i=p+1}^{d} \lambda_i)/(d-p)$ (see the Supplementary Information). The concavity of the logarithm implies that the empirical entropy decreases monotonically with p (and, incidentally, that the larger the heterogeneities of $\boldsymbol{\lambda}$, the lower the empirical entropy).

4. PCA induces a multivariate normal distribution whose average vector $\boldsymbol{\mu}$ is the unbiased estimator of this quantity in the dataset $\mathcal{I}_{\mathrm{tr}}$, and whose covariance matrix C shares the p largest-eigenvalues and corresponding eigenvectors with the sample covariance matrix of the set $\mathcal{I}_{\mathrm{tr}}$ (see the details in the Supplementary information).

5. Eigenface coding is usually understood as a representation in terms of non-local principal components. Please note that it would be misleading to interpret the decoupled coding $\mathcal{R}_{\mathrm{D}}$ as being local instead, just because it uses landmark coordinates. Indeed, $\mathcal{R}_{\mathrm{E}}$ represents facial images in terms of principal components $\mathbf{I}'$, and so does $\mathcal{R}_{\mathrm{D}}$: $\hat{\mathbf{I}}'$ are non-local in the sense that each component is a linear combination of pixel intensities occupying different positions in the image canvas; $\boldsymbol{\ell}'$ is non-local as well, in the sense that each component is a linear combination of different landmarks' Cartesian coordinates.

6. Note that, crucially, this approach is different from a standard *Bayesian model selection*, in which different models are compared using the same dataset; see more details in the Supplementary information, section *Relation with Bayesian Model Selection.*

7. In other words, if one assumes that the pixel values are uniformly distributed around their average in a d_{t}-dimensional hypercube of size $R = (12\bar{v}_{\mathrm{t}})^{1/2}$, then $l_0/dN = (1/2)\log_2(12\bar{v}_{\mathrm{t}}) - \log_2 \epsilon$. This value is very close to the empirical entropy of the dataset corresponding to a PCA model with $p = 0$ (see the Supporting information): $L_0 = \mathsf{S}_0 = (1/2)\{\log_2(2\pi) + 1 + \log_2(\tilde{v})\} - \log_2 \epsilon$, where $\log_2 \tilde{v} = \overline{\log_2 \lambda}$ (see the proximity of L_0 and l_0 in Fig 2 of the main text).

8. In the language of probability, we have seen before that the uniformed images present stronger between-pixel correlations C_{ij} while presenting a roughly equal total variance (or $\mathrm{tr}\,(C)$). This is the reason for which, for uniformed faces, the training-set empirical entropy ($\sum_i \ln \lambda_i$, up to a constant) is lower (hence the likelihood is higher). A lower test-set entropy would simply imply that also the term $\mathrm{tr}\,(C_{\mathrm{te}} \cdot C_{\mathrm{tr}}^{-1}/2)$ (the difference between test- and training-set entropies, up to a constant) is significantly lower. We will call bias and variance terms of the entropy to the terms $\sum_i \ln \lambda_i$ and $\mathrm{tr}\,(C_{\mathrm{te}} \cdot C_{\mathrm{tr}}^{-1}/2)$, respectively.

9. In particular, we sample 20 principal components x'_i from their respective distributions, where the index i may take the values $1, \ldots, p = 40$. The remaining 20 coordinates among the first 40 coordinates are set to zero.

10. Since $d_{\mathrm{t}} \gg d_{\mathrm{s}}$, and since, as we mention in the Supplementary information, both coordinates are weakly correlated, the eigenvalues of $C^{(\mathrm{c})}$ are dominated by those of $C^{(\mathrm{t})}$ and the likelihoods almost coincide. For this reason, and because of the lack of a biological motivation, we do not present an information-theoretical analysis of the concatenated model.

11. Although surely not the most efficient method for a supervised classification analysis, we choose the Mahalanobis distance algorithm, since it is the one that uses the only the information defining our working models, i.e., C_p for each kind of coordinate.

12. $\mathsf{d}_p(\mathbf{u}, \mathbf{v}) = [(\mathbf{u} - \mathbf{v})^\dagger \cdot C_p^{-1} \cdot (\mathbf{u} - \mathbf{v})]^{1/2}$, where $C_p = E_p^\dagger \cdot \Lambda_p \cdot E_p$, and where Λ_p is the diagonal matrix of the largest p eigenvalues.

13. If $\mathbf{x}$ corresponds to an image, the reconstructed image is different from the original one even with $p = N$ coordinates, since the matrix $E_p^\dagger \cdot E_p$ is different from the identity matrix, as far as it has rank $= p \leq N < d_{\mathrm{t}}$.

Relation with Bayesian Model Selection. Bayesian model selection consists in choosing the model $\mathcal{M}$ that maximises the Bayesian evidence of a given dataset $\mathcal{D}$. The best model $\mathcal{M}$ is, equivalently [2], the one that minimises the description length

$\min_{\mathcal{M}} L_{\mathcal{M}}(\mathcal{D})$. To verify the validity of the condition in Eq. (3) in the main text, we have, instead, compared the description length of *different datasets*: $\mathcal{I}$, $\hat{\mathcal{I}}$, $\mathcal{L}$, according to *the same probabilistic model*, which corresponds to the multivariate normal distribution whose correlation matrix takes, respectively, the values C_p, $C^{(\mathrm{t})}_{p_\mathrm{t}}$ and $C^{(\mathrm{s})}_{p_\mathrm{s}}$, for the three datasets. Here, C_p is the matrix whose p largest eigenvalues and corresponding eigenvectors are the same of the sample eigenvalues and eigenvectors of the training-set of non-uniformed images and the remaining $d-p$ eigenvalues are set to a constant (see, for example, [3]), and the same for $C^{(\mathrm{t})}_{p_\mathrm{t}}$ and $C^{(\mathrm{s})}_{p_\mathrm{s}}$.

This is, hence, the opposite situation with respect to Bayesian model selection, in which one compares the evidence of the same dataset according to different models. It is important to remark that, in the present work, we do not aim to perform a comparison, on Bayesian grounds, between eigenface and decoupled codings, understood as probabilistic models *over the common dataset of original, non-uniformed facial images.* Indeed, the representation $\mathcal{R}_\mathrm{D}$ induces a probability distribution in the space of non-uniformed images $\mathbf{I}$, that is no longer a Gaussian distribution (even if the distributions over $\hat{\mathbf{I}}$ and $\boldsymbol{\ell}$ are) since it involves the nonlinear image deformation operations, that we have completely neglected in our information-theoretical analysis. Within our working hypothesis, we neglect the *uniformation* $\mathcal{I}, \mathcal{L} \to \hat{\mathcal{I}}$ (previous to the PCA) and *de-uniformation* $\mathcal{I}_p, \mathcal{L}_p \leftarrow \hat{\mathcal{I}}', \mathcal{L}'$ (posterior to the PCA) operations from the information-theoretical analysis.

In other words, here we consider three probabilistic descriptions over separate spaces: non-uniformed images, uniformed images and shape coordinates. Our conclusions merely rely in the information-theoretical interpretation of the Bayesian evidence of a dataset according to a model, that is related to the amount of information needed to store the dataset in terms of the model lattent variables, with a given precision. The decoupled code, understood as a probabilistic model *on the space of the original images* would be more complex than Gaussian. It would implicitly contain, in some (texture) latent variables, a description of the input image somehow invariant under shape transformations; other (shape) latent variables would be invariant under texture transformations of the original image. Our current analysis is to be understood as an estimation of the information theoretical gain of the facial representation in terms of (principal components of) uniformed images and landmarks, neglecting the non-linear operations of landmark detection and image deformation that lead to these two facial coordinates, from the original dataset of images.

Instead, we do perform a genuine Bayesian model selection when choosing the values of p, p_t, p_s that minimise the description length (that maximise the Bayesian evidence) of each dataset, i.e., of each type of coordinate.

Image uniformation and de-uniformation. The creation of the *uniformed* texture $\hat{\mathbf{I}}$ coordinates from the original images $\mathbf{I}$ and their shape coordinates $\boldsymbol{\ell}$ in $\mathcal{R}_\mathrm{D}$ is implemented, as said before, through image deformation algorithms based on similiarity transformations [4]. Such algorithms map the original image into an image whose landmark positions $\boldsymbol{\ell}$ will now occupy their average value in the dataset $\langle\boldsymbol{\ell}\rangle$. Vice-versa, the reconstruction of novel images in $\mathcal{R}_\mathrm{D}$ requires creating a non-uniformed facial image from the reconstructed shape and texture coordinates $\boldsymbol{\ell}_p, \hat{\mathbf{I}}_p$. This operation we will be called *de-uniformation*:

$$(\boldsymbol{\ell}, \mathbf{I}) \quad \overset{\langle\boldsymbol{\ell}\rangle}{\to} \quad (\langle\boldsymbol{\ell}\rangle, \hat{\mathbf{I}}) \qquad \text{uniformation} \tag{1}$$

$$(\boldsymbol{\ell}, \mathbf{I}) \quad \overset{\boldsymbol{\ell}}{\leftarrow} \quad (\langle\boldsymbol{\ell}\rangle, \hat{\mathbf{I}}) \qquad \text{de-uniformation} \tag{2}$$

where the subscripted arrow indicates the image deformation algorithm transforming an image $(\boldsymbol{\ell}_1, \mathbf{I}_1) \overset{\boldsymbol{\ell}_2}{\to} (\boldsymbol{\ell}_2, \mathbf{I}_2)$ so that the pixel values of $\mathbf{I}_2$ in the positions given by $\boldsymbol{\ell}_2$ are

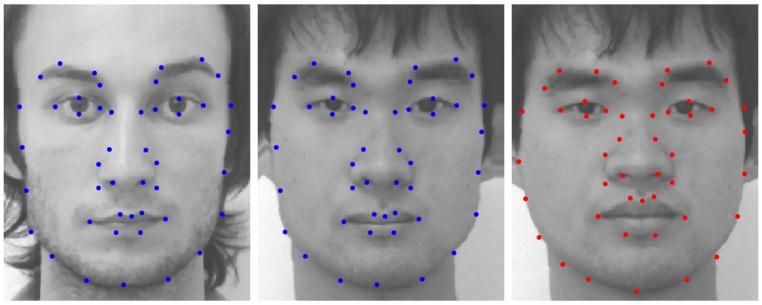

Fig 1. an example of usage of the software for image deformation. The image in the center is the image deformation of the right image with the landmarks corresponding to the left image. The original images (left and right) are republished from [5] under a CC BY license, with permission from Carlos Eduardo Thomaz, original copyright 2006.

those of $\mathbf{I}_1$ in $\boldsymbol{\ell}_1$ (say, $\mathbf{I}_2(\vec{\ell}_{2j}) = \mathbf{I}_1(\vec{\ell}_{1j})$ where $\vec{\ell}_{1j}$ are the original Cartesian positions of the j-th landmark), and the rest of the pixel values of $\mathbf{I}_2$ are changed consequently, under the requirement of smoothness. As a consistency check, we have verified that uniforming and consequently de-uniforming dataset images, leads to new images that are visually indistinguishable from the initial ones.

In fig.1 we illustrate the effect of the used image deformation algorithm on a picture of the FEI database.

How many landmarks are too many? The results presented in the main text lead to a picture of the origin of the efficiency of the decoupling code $\mathcal{R}_{\mathrm{D}}$. In substance, the above analysis suggests that, for the decoupling to be worth, *the number of landmarks should be low enough, relatively to the number of pixels.* In this situation, few landmarks, of the order of some tenths, require few information to be encoded and, at the same time, they imply a large texture information gap in since the resulting uniformed images are more compressible. Increasing the number of landmarks would increase their description length $\mathcal{G}_2$ and, at the same time, it would entail a lower and lower increase in the texture information gap $\mathcal{G}_1$, eventually leading to a negative overall gap $\mathcal{G}$. Roughly speaking, if the landmarks were too much given the image resolution, they would encode information regarding the details of the shape that is already present in the original images, and that does not further help compressing them when uniformed consequently.

In the following two paragraphs we discuss the the role plaid by d_{s} and d_{t}. On the one hand, the texture coordinates description length $\mathcal{G}_1$ increases with the number of pixels d_{t}. The description length of the shape coordinates $\mathcal{G}_2 = L_{\mathcal{L}_{\mathrm{tr}},p_{\mathrm{s}}}(\mathcal{L}_{\mathrm{tr}})$ increases as well with the number of landmark coordinates d_{s}.[1] In both cases, however, the dependence quickly becomes linear in d: respectively, when d_{t} approaches the finer resolution (in our dataset, $h = 300$), and when the number of landmarks is so large that the average distance between landmarks becomes of the order of the image grid space unit (or much sooner).

On the other hand, the texture information gap $\mathcal{G}_1$ itself increases *with the number*

[1] Both dependences are slightly over-linear for lower values of d_{s} and d_{t}. In the case of texture coordinates, this is the over-linear behaviour that we observed in figure 2, main text. Analogously, adding landmark coordinates $d'_{\mathrm{s}} > d_{\mathrm{s}}$ will lead to a shape description length slightly larger than $(d'_{\mathrm{s}}/d_{\mathrm{s}})\mathcal{G}_2$, since a finer description of the facial shape contour will reveal information beyond what can be deduced deterministically (e.g., by interpolation) from the coarser description of each image's shape in terms of a lower number of landmarks.

of landmarks.[2] However, when the average inter-landmark distance becomes of the order of the grid space unit, $d_{\rm s}$ has no longer impact on $\mathcal{G}_1$. Increasing the number of landmarks will not influence the texture information gap.

The two above straightforward arguments motivate an order of magnitude estimation of the maximum shape dimension $\bar{d}_{\rm s}$ (twice the maximum number of landmarks) beyond which the uniformation is no longer worth, for the largest of the considered resolutions, $h_{\max} = 300$. Such an estimation is based on neglecting the over-linear dependence of $\mathcal{G}_2$ on $d_{\rm s}$ and the dependence of $\mathcal{G}_1$ on $d_{\rm s}$.

The decoupling is efficient in the extent to which the information gap is large, and larger than zero. Under the above hypotheses, $\bar{d}_{\rm s}$ is consequently the dimension by which one must multiply the shape description length per coordinate in order to reach $\mathcal{G}_1$ (see figure 2 in the main text, and *Likelihood and evidence of shape coordinates*): it is $\bar{d}_{\rm s} \simeq \mathcal{G}_1 d_{\rm s}/L_{\mathcal{L}_{\rm tr},p_{\rm s}}(\mathcal{L}_{\rm tr})$, or $\bar{d}_{\rm s} \simeq 1700 \pm 100$, roughly less than one thousand landmarks.

We notice, however that, under these crude hypotheses, the information gap monotonically decreases with $d_{\rm s}$. The more interesting estimation not of the maximum but of the *optimal* number of landmarks, that maximises the information gap for a fixed image resolution, would require to take into account the non-linear dependences on $d_{\rm s}$, neglected in the above argument (see footnote 2). We expect, in any case, that such an optimal value would be much lower than the above estimation for its maximum value, probably of the order of few hundred of landmarks, for $h \simeq 300$.

Likelihood and evidence of the normal distribution. We report the well-known expression for the Bayesian evidence, and related formulae, of the normal distribution associated to the p-PCA representation with p principal components. Given a dataset $\mathcal{D}$ composed by N d-dimensional vectors, p-PCA induces a likelihood probability distribution which is the normal distribution (supposing null averages):

$$\ln P(\mathcal{D}|\boldsymbol{\theta}, p)/(Nd) = -\frac{1}{2}\left[\ln(2\pi) + \ln\det(C) + \mathrm{tr}\left(C^{-1}\cdot\Sigma\right)\right] \tag{3}$$

where Σ is the unbiased estimator of the correlation matrix of the data $\mathcal{D}$, and where the parameters $\boldsymbol{\theta} = C$ are the theoretical correlation matrix, which in p-PCA is subject to exhibit its $d-p$ lowest eigenvalues equal to a common noise-level value v. The maximum likelihood estimation $\boldsymbol{\theta}^*$ for C and v are: $C^* = U\Lambda U^\dagger$ where U is an orthogonal matrix whose top p eigenvectors are those of Σ, and where the diagonal matrix Λ contains the top p eigenvalues of Σ, $\Lambda_{ii} = \lambda_i$ for $i \le p$, and the remaining $d-p$ diagonal elements equal to $\Lambda_{ii} = v_p$ for $i > p$, with $v_p = (d-p)^{-1}\sum_{j>p}\lambda_j$.

For completeness, we report the expressions for the description length, the empirical entropy and the Occam factor, making explicit the dependence on the number of principal components p:

$$L_p(\mathcal{D}) = -\ln P_p(\mathcal{D}) - d\ln\epsilon = \tag{4}$$

$$= \mathsf{S}_p(\mathcal{D}|\boldsymbol{\theta}^*) + \mathsf{O}(\boldsymbol{\theta}^*) \tag{5}$$

$$\mathsf{S}_p(\mathcal{D}|\boldsymbol{\theta}) = -\ln P_p(\mathcal{D}|\boldsymbol{\theta}) - d\ln(\epsilon) \tag{6}$$

$$\ln P_p(\mathcal{D}) = \ln P_p(\mathcal{D}|\boldsymbol{\theta}^*) - \mathsf{O}_p(\boldsymbol{\theta}^*) \tag{7}$$

[2] We do not present such an analysis since a systematic exploration is limited in a dataset with moderate number of landmarks. For n_ℓ of the order of a dozen, the resulting description length critically depends on *which landmarks* are considered. Furthermore, the image deformation algorithms (needing a minimally dense mesh of landmarks covering the non-uniform parts of the images) necessarily induce artefacts in the uniformed images for low n_ℓ and, consequently, in the description lengths. Rather than a numerical estimation in our dataset of limited $d_{\rm s}$, we find clearer the order-of-magnitude argument presented in this paragraph.

where, as mentioned in the main text, in these equations $\boldsymbol{\theta}^*$ refers to the maximum likelihood estimator. The equation for the Bayesian evidence (under certain assumptions on the prior variance) takes the form, up to a constant factor, and for sufficiently large N [3]:

$$\ln P(\mathcal{D})/(Nd) \simeq \ln P(\mathcal{D}|\boldsymbol{\theta})/(Nd) - \ln \mathsf{O}(\boldsymbol{\theta}^*)/(Nd) \tag{8}$$

$$-\ln \mathsf{O}(\boldsymbol{\theta}^*)/(Nd) := \frac{1}{2Nd}\left((m+p+1)\ln(2\pi) - p\ln N - \ln|A| + \ln|p_U|\right) \tag{9}$$

$$m := dp - p(p+1)/2 \tag{10}$$

$$\ln|p_U| := -p\ln 2 + \sum_{j=1} p \ln\Gamma\left(\frac{d-j+1}{2}\right) - \frac{d-j+1}{2}\ln\pi \tag{11}$$

and where:

$$\ln|A| = \sum_{i=1}^{p}\left\{\sum_{j=i+1}^{d}\left[\ln(\hat{\lambda}_j^{-1} - \hat{\lambda}_i^{-1}) + \ln(\lambda_i - \lambda_j)\right]\right\} + m\ln N \tag{12}$$

where the λ's are the eigenvalues of Σ in decreasing order, $\hat{\lambda}_j = \lambda_j$ for $j \leq p$ but $= v_p$ for $j > p$.

In the case $d > N$, this last term takes the form:

$$\ln|A| = \sum_{i=1}^{p}\left\{\sum_{j=i+1}^{p}\left[\ln(\lambda_j^{-1} - \lambda_i^{-1}) + \ln(\lambda_i - \lambda_j)\right] + \right. \tag{13}$$

$$+ (d-p)\ln(v_p^{-1} - \lambda_i^{-1}) + \sum_{j=p+1}^{N}\ln(\lambda_i - \lambda_j) + \tag{14}$$

$$\left. + (d-N)\ln\lambda_i \right\} + m\ln N \tag{15}$$

while for $d \leq N$, it is:

$$\ln|A| = \sum_{i=1}^{p}\left\{\sum_{j=i+1}^{p}\left[\ln(\lambda_j^{-1} - \lambda_i^{-1}) + \ln(\lambda_i - \lambda_j)\right] + \right. \tag{16}$$

$$\left. + (d-p)\ln(v_p^{-1} - \lambda_i^{-1}) + \sum_{j=p+1}^{d}\ln(\lambda_i - \lambda_j)\right\} + m\ln N. \tag{17}$$

Likelihood and evidence of shape coordinates. For shape coordinates, and for the datasets considered here, it is $d_{\mathrm{s}} < N$. In figure 2 (upper panel) we show the behaviour of the training- and test-set (logarithms of the) likelihood, along with the training- and test-set (logarithms of the) Bayesian evidence of shape coordinates (respectively, $\ln P(\mathcal{L}_{\mathrm{tr}}|C^{\mathrm{s}})$, $\ln P(\mathcal{L}_{\mathrm{te}}|C^{\mathrm{s}})$, $\ln P(\mathcal{L}_{\mathrm{tr}})$, $\ln P(\mathcal{L}_{\mathrm{te}})$). We observe that the training-set evidence behaviour is qualitatively similar to that of the the test-set likelihood (contrary to the case of texture coordinates, see below).

When commenting the results of figure 3 in the main text, we mentioned the fact that the empirical entropy of shape coordinates does not depend on the resolution. Indeed, changing the resolution in the dataset of shape coordinates amounts to multiply

the Landmarks' Cartesian coordinates by a factor (w/w' for horizontal, h/h' for vertical coordinates). However, the relevant quantity in these experiments is not the absolute value of the coordinates in the $w \times h$ grid units, but their normalised value in units of the image heigh h. If normalised coordinates are considered, the precision should be consequently normalised to be inversely proportional to h. In figure 2 (lower panel) we plot the training empirical entropy $S_p(\mathcal{L}_{\rm tr}|C^{\rm s}) = -\ln P(\mathcal{L}_{\rm tr}|C^{\rm s}) - d_{\rm s} \ln \epsilon_{\rm s}$ for different resolutions, using the resolution-dependent precision $\epsilon_{\rm s} = 0.1(h_{\rm max}/h)$. The overlap of different curves is a consequence of the fact that no information has been lost when scaling both the coordinates and the precision.

Likelihood and evidence of texture coordinates. In figure 3 we show the behaviour of the training- and test-set (logarithms of the) likelihoods along with the training- and test-set (logarithms of the) Bayesian evidence of texture coordinates. In this case, in which, differently from shape coordinates, it is $d_{\rm t} \gg N$, the BIC approximation for the evidence is, as expected, no longer good. Moreover, the evidence behaves differently from the test-set likelihood. In order to perform model selection in this case, or to estimate the Occam contribution to the description length, it is necessary to use the aforementioned expression of the Bayesian evidence due to Minka.

The reader may wander why the texture coordinates' seem to present strong overfitting in figure 3, where the test-likelihood presents a fast decreasing for $p \gtrsim 100$, while the opposite seems to hold in the facial recognition task, figure 6 in the main article, where the shape coordinates present a minimum of the classification error rate for $p \gtrsim 30$, while texture coordinates do not seem to overfit in this case. On the one hand, in the case of figure 3, this is what one expects in a situation in which $d \gg N$. In this situation, the training-set variance of the p-th modes λ_p are underestimated (see, for example [6]) and, consequently, the negative test-set *energy* (or minus the exponent of the normal distribution probability density, $\mathrm{tr}\,(C_{\rm te} \cdot C_{\rm tr}^{-1}) = \sum_k^p \langle {x'_k}^2 \rangle_{\rm te}/\lambda_k$) increases fast with p. This is the difference between the test and training log-likelihood. Such a difference is also present for shape coordinates in figure 2, in this case the training-test difference in likelihood is lower (and roughly equal to $-m\ln(N)/2N$, since Minka and BIC evidences coincide), meaning that also in this case x'_k varies more than $\lambda_k^{1/2}$ in the test-set.

On the other hand, for the classification error, the (squared) Mahalanobis similarity between the s-th and s'-th subjects is:

$$\sum_{j=1}^{p} \frac{1}{\lambda_j} \left(y'_j(s, \text{smile}) - y'_j(s', \text{neutral}) \right)^2 \qquad (18)$$

The difference, in this case, is that shape coordinates vary drastically between smile and neutral facial images, and are not that characteristic of the subject's identity as texture coordinates are. The high-j terms in the above equation present strong random fluctuations even if $s' = s$, since high-j fluctuations y'_j are not representative of the subject, and are over-weighted by the relatively smaller λ_j. This is the origin of the increasing of the error rate with p. The same over-weighting of the fluctuations happens for texture coordinates, although, in this case, $D_{s,s',j} = y'_j(s, \text{smile}) - y'_j(s', \text{neutral})$ is still more similar when $s' = s$ than for $s' \neq s$ even for hight j, *and/or* $D_{s,s',j}$ is so low for low values of j, that it compensates the overall increase in $D_{s,s',j}$ for larger values of j. The fact that the error rate does not seem to increase at all with p in figure 6, main text, suggests that the first of the two hypothesis in the above sentence holds.

The concatenated code representation, $\mathcal{R}_{\rm c}$ (see figure 4), simply consists in concatenating the uniformed texture and shape coordinates in a single vector $\mathbf{y} = (\boldsymbol{\ell}, \hat{\mathbf{I}})$,

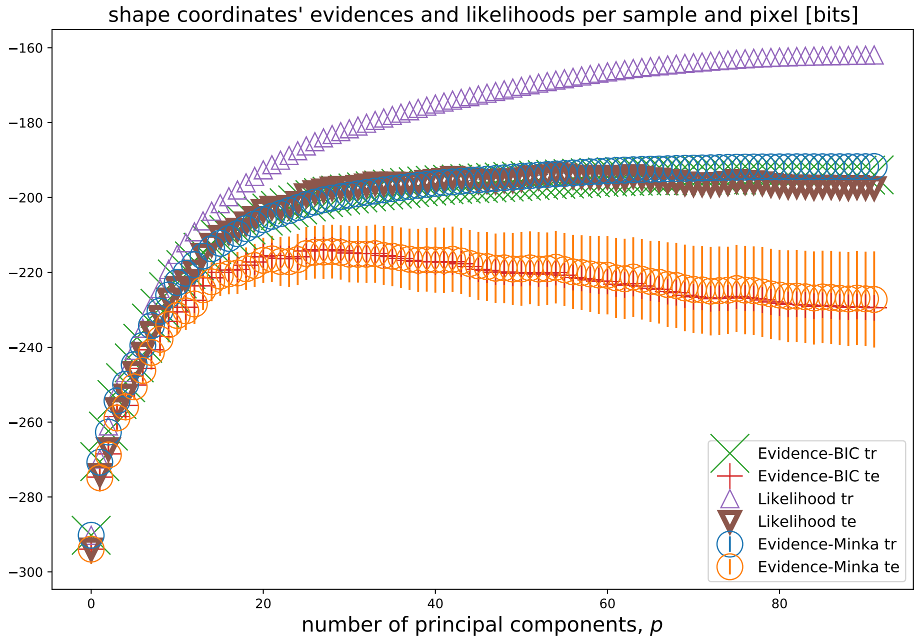

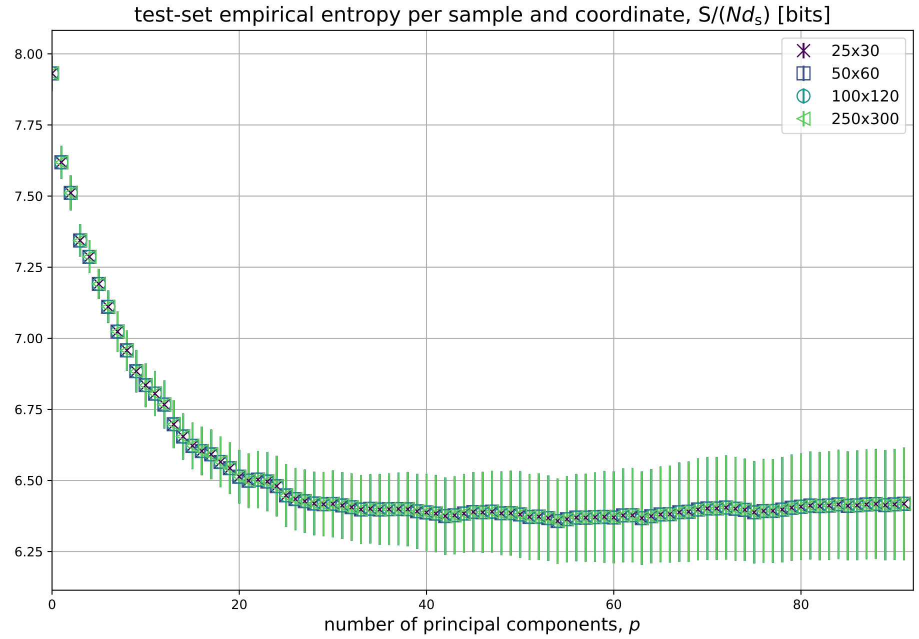

Fig 2. Left: Shape coordinates' likelihoods and evidences (in BIC approximation) of the test and training sets. Right: Empirical entropy of shape coordinates for the full $N_{\mathrm{tr}} = 400$ training set, for different resolutions.

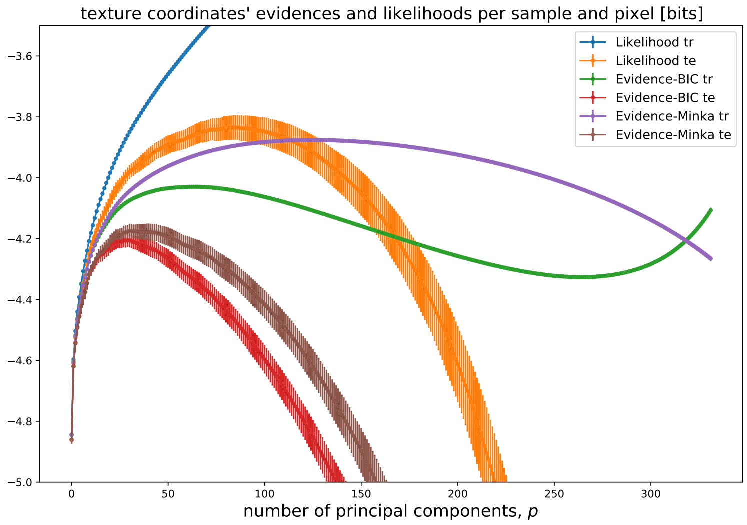

Fig 3. Uniformed texture coordinates' likelihoods and evidences of the test and training sets.

and to keep the first p_{c} principal components of the set of concatenated vectors $\mathbf{y}' = E^{(\mathrm{c})}\mathbf{y}$, hence treating shape and texture coordinates on the same ground. For an image dataset such that shape and texture coordinates were completely uncorrelated (say, $\langle \ell_m I_i \rangle = 0 \;\forall m, i$), the concatenated code would exactly coincide with $\mathcal{R}_{\mathrm{D}}$, in the sense that each principal axis would be a (normalised) concatenation of principal axes of $\boldsymbol{\ell}$ and $\hat{\mathbf{I}}$ coordinates. The performance of the $\mathcal{R}_{\mathrm{c}}$ code in the face processing tasks presented in section Results turns to be almost identical using texture coordinates only. The reason is that shape coordinates carry a lower amount of aggregated information and, in any case, the correlations between shape and texture coordinates are significantly smaller than those in the diagonal blocks of $C^{(\mathrm{c})}$. The advantage of using $\mathcal{R}_{\mathrm{c}}$ is that one may fix a single number of principal components. The daydream generation of novel facial images with the $\mathcal{R}_{\mathrm{D}}$ code (fixing $p_{\mathrm{s}} = d_{\mathrm{s}}$ at its maximum value) leads to almost identical results of those of $\mathcal{R}_{\mathrm{c}}$ in figure 5 in the main text.

Details of the classification algorithms. The classification tasks are performed via a nearest-neighbour classifier: every vector $\mathbf{x}$ is assigned to the class that minimizes the distance from $\mathbf{x}$. If a class contains more than one element, as it is the case of the gender classification task (in which the male and the female classes contain 200 vectors each, corresponding to half of the raw FEI database), the distance is computed between $\mathbf{x}$ and the average of the elements belonging to the class. For the gender identification task we follow a leave-one-out approach: for each vector $\mathbf{x}$, the training-set (from which we compute the correlation matrix, defining in its turn the Mahalanobis distance $\mathsf{d}_p(\cdot,\cdot)$) is composed by all the dataset vectors except for $\mathbf{x}$ itself. The so defined training-set is as well the set from which the average vector of each class is constructed.

In the facial recognition task, we use a more economic strategy: we construct $K = 5$ training/test-set divisions (with $N_{\mathrm{te}} = 400/K = 80$, $N_{\mathrm{tr}} = 320$) by K-folding, in such a way that the test-set contains at most one vector per individual, and that it contains $N_{\mathrm{te}}/2$ vectors corresponding to smiling portraits, and $N_{\mathrm{te}}/2$ corresponding to neutral portraits. For each of the vectors of facial coordinates in the test-set, we search for its

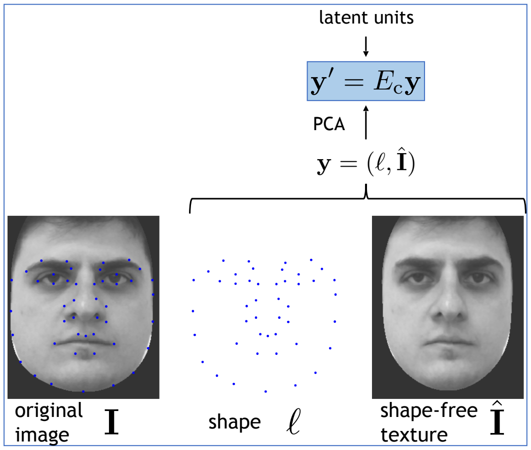

Fig 4. Schematic representation of the concatenated code. The original image (left) is republished from [5] under a CC BY license, with permission from Carlos Eduardo Thomaz, original copyright 2006.

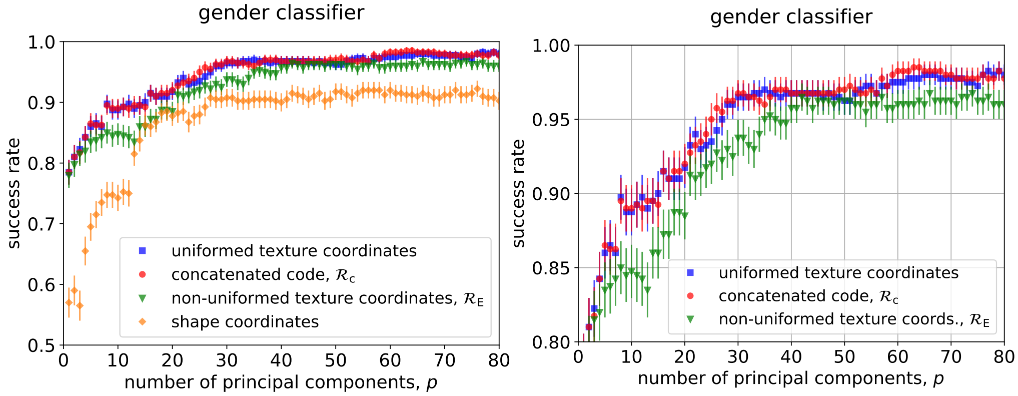

Fig 5. Success rates in the task of gender classification as a function of the number P of principal components; in the right column the same data (except for the geometric coordinates) are shown in a close-up.

nearest neighbor among the $N_{\rm tr} = 320$ vectors of facial coordinates in the training set. The training set is, again, the set from which we compute C_p and consequently define $\mathsf{d}_p(\cdot,\cdot)$. Afterwards, the average value and the standard deviation of the mean of the success rate is computed by cross-validation over the $K = 5$ iterations.

Results of the gender classification task. In figure 5 we present the results of the gender classification task. We observe that the shape coordinates alone are sufficient to achieve roughly 90% of successful attempts with less than 30 PC's. Consistently with the rest of the article results, the classification performed in terms of (principal components of) uniformed facial images achieves higher success rates respect to that using (principal components of) the original original facial image (i.e., the $\mathcal{R}_{\rm E}$ representation). Furthermore, the success rate plateau is reached for a lower number of PC's ($p \simeq 30$ versus $p \simeq 40$ of $\mathcal{R}_{\rm E}$).

Different regularisation schemes. For each generic set of facial coordinates (say, $\mathcal{D}$), we have so far estimated its description length according to the p-PCA model,

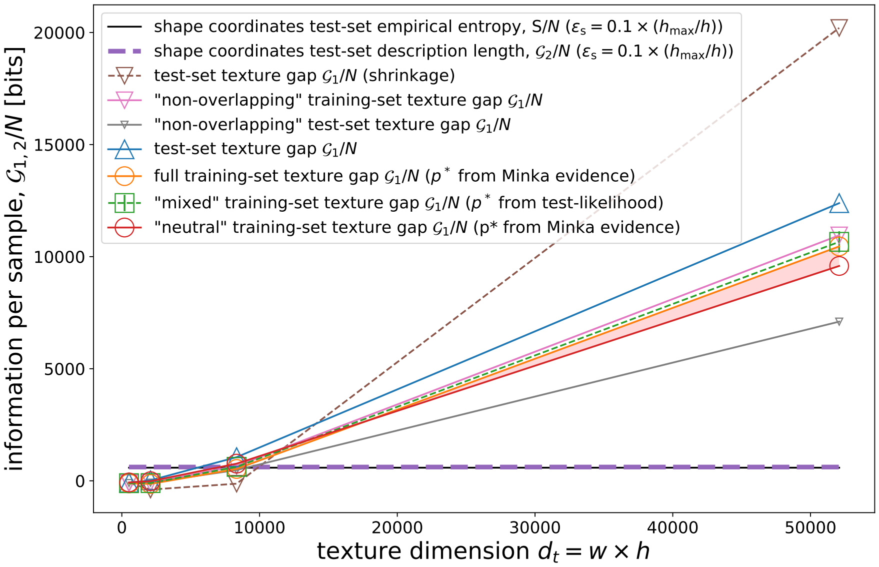

Fig 6. The same as figures 3, 4 in the main text, but with the addition of the test-set texture gap per sample $\mathcal{G}_1/N$ computed with the shrinkage regularisation method.

whose number of principal components p are those that maximise the description length $p^* = \arg\min_p L_{\mathcal{D},p}(\mathcal{D})$. The inferred probability distribution is a normal distribution whose correlation matrix C_{p^*} is consequently different from the empirical correlation matrix, say $C_{p=\min\{N,d\}}$, since not all empirical eigenvalues and eigenvectors are statistically significant given the dataset finiteness.[3] The normal distribution whose correlation is the empirical matrix would correspond, instead, to maximum likelihood inference.

Actually, there are different ways, besides p-PCA, in which the correlation matrix may be inferred beyond the maximum likelihood criterion. An alternative is *linear (identity) shrinkage* (see, for example, [6]). Linear shrinkage leads to a correlation matrix which is a convex combination between the unbiased (maximum likelihood) empirical estimator C and a completely biased (and null-variance) matrix, as the identity matrix in d dimensions 1_d. In other words, the "regularised" shrunk matrix is $C_\alpha = (1-\alpha)C + \alpha 1_d$ where α is a real number in $[0,1]$, that may be chosen by maximum (cross-validated) out-of-sample likelihood. In the p-PCA scheme, $p = 0$ and $p = \min\{N,d\}$ are the arguments of the minimum and maximum training likelihood respectively, and p^* is comprised between them. Within the shrinkage scheme, these extreme cases correspond to $\alpha = 1$ and 0, respectively.

In order to check the robustness of our results with respect to the regularisation scheme, we have computed the information gaps (actually, the gaps in empirical entropy)[4] resulting from the normal probability distributions associated not with p-PCA but with linear shrinkage. We have observed that the results are qualitatively consistent

[3]Strictly speaking, in the $N < d$ case, the inferred correlation matrix C_p is different from the empirical matrix even if $p = N$, since it has to be regularised so that its rank is $= d$ (and not $= N$).

[4]We make notice that, in the case of p-PCA, and for texture coordinates, the texture gap $\mathcal{G}_1$ is essentially given by the gap between empirical entropies. The difference between the Occam factors of non-uniformed and uniformed images is negligible in front of it.

with those presented here. While the lowest description length of the set of landmarks $\mathcal{G}_2$ is consistent with the one shown in figure 3, main text, the description length gap $\mathcal{G}_1$ is significantly larger for the largest resolution, as can be seen in figure 6. Consequently, the information gap is even larger when regularising the correlation matrices with the shrinkage method.

Visualisation of the eigenvectors of the concatenated code $\mathcal{R}_c$. Figure 7 presents a graphical visualisation of the first five principal axes of the whole database according to the concatenated code $\mathcal{R}_c$ (the first five eigenvectors of $C^{(c)}$).

References

1. Chang L, Tsao DY. The Code for Facial Identity in the Primate Brain. Cell. 2017;169(6):1013–1028.e14. doi:10.1016/j.cell.2017.05.011.

2. MacKay DJ, Mac Kay DJ. Information theory, inference and learning algorithms. Cambridge university press; 2003.

3. Minka TP. Automatic choice of dimensionality for PCA. In: Proceedings of the 13th International Conference on Neural Information Processing Systems; 2000. p. 577–583.

4. Schaefer S, McPhail T, Warren J. Image Deformation Using Moving Least Squares. In: ACM SIGGRAPH 2006 Papers. SIGGRAPH ’06. New York, NY, USA: Association for Computing Machinery; 2006. p. 533–540. Available from: `https://doi.org/10.1145/1179352.1141920`.

5. FEI Face Database;. `https://fei.edu.br/~cet/facedatabase.html`.

6. Bun J, Bouchaud JP, Potters M. Cleaning large correlation matrices: Tools from Random Matrix Theory. Physics Reports. 2017;666:1–109. doi:https://doi.org/10.1016/j.physrep.2016.10.005.

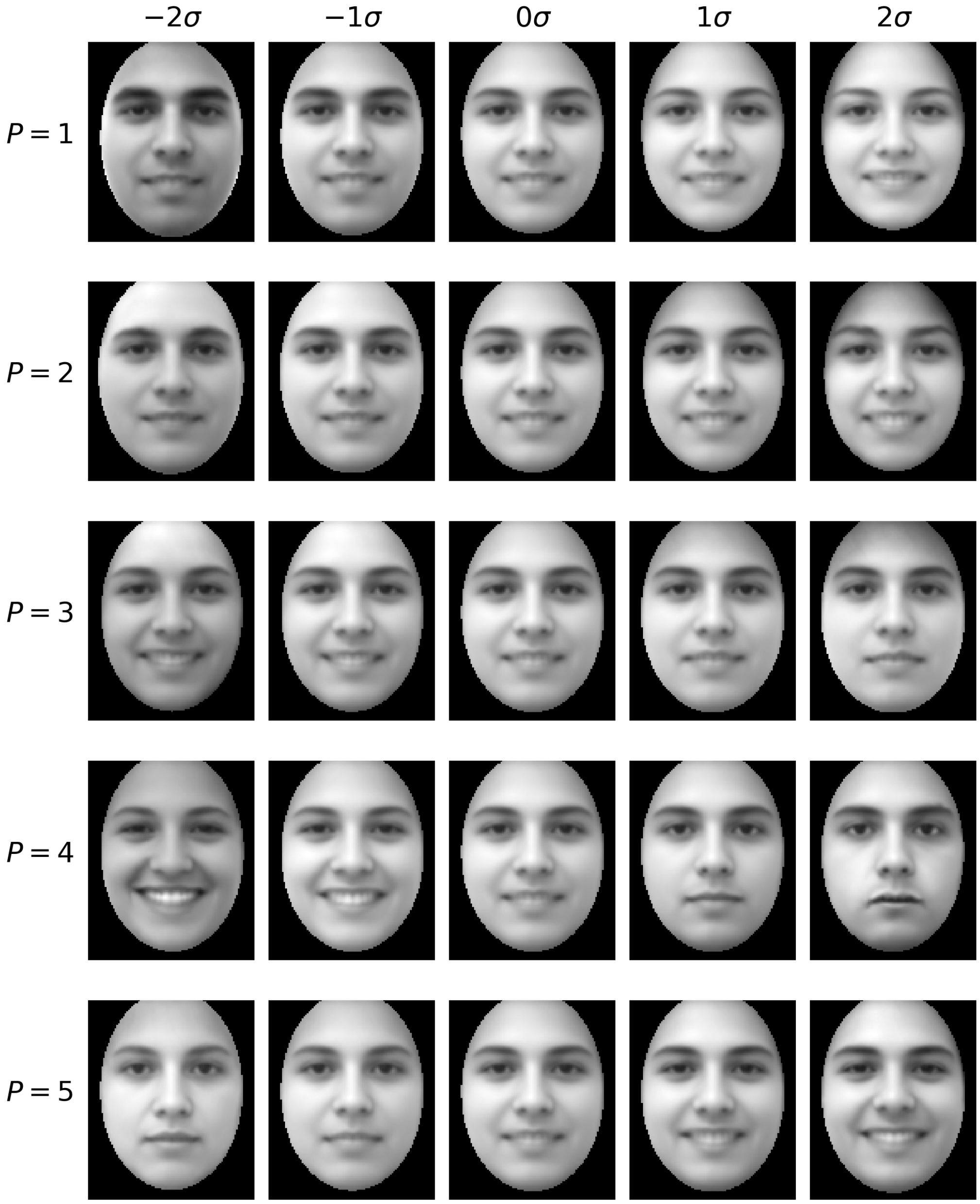

Fig 7. First five principal axes of the concatenated code $\mathcal{R}_{\rm c}$ (five largest-eigenvalue eigenvectors of $C^{(\rm c)}$). The j-th column represents the $j = th$ eigenvector. In particular, the i-th row of the table represents the points that have all the coordinates,in the base of the principal axes, equal to zero except the i-th one, that ranges from -2σ (left) to -2σ (right); σ is taken equal to the square root of the largest eigenvalue λ_1 of the correlation matrix. In other words, the image, say $\mathbf{I}$ in the i-th row, j-th column is obtained by de-uniformation $(\langle \boldsymbol{\ell} \rangle, \hat{\mathbf{I}}) \to (\boldsymbol{\ell}, \mathbf{I})$, where $\boldsymbol{\ell}$ and $\hat{\mathbf{I}}$ are obtained as: $(\boldsymbol{\ell}, \hat{\mathbf{I}}) = \mathbf{y} = E^{\dagger} \cdot \mathbf{y}'$, and where $\mathbf{y}'$ is the vector that exhibits null principal components except by the i-th, $y'_i = (j-3)\lambda_1^{1/2}$, and E is the matrix of row eigenvectors of $C^{(\rm c)}$.
